# Quantifying spectral changes experienced by plasmonic nanoparticles in a cellular environment to inform biomedical nanoparticle design

**DOI:** 10.1186/1556-276X-9-454

**Published:** 2014-08-31

**Authors:** Allen L Chen, Ying S Hu, Meredith A Jackson, Adam Y Lin, Joseph K Young, Robert J Langsner, Rebekah A Drezek

**Affiliations:** 1 Department of Bioengineering, Rice University, Houston, TX 77005, USA; 2 Waitt Advanced Biophotonics Center, Salk Institute for Biological Studies, La Jolla, CA 92037, USA; 3 Department of Electrical and Computer Engineering, Rice University, Houston, TX 77005, USA

**Keywords:** Nano-bio interactions, Plasmon resonance, Hyperspectral imaging, Gold nanoparticles, Cells, Spectral analysis, Nanomedicine

## Abstract

Metal nanoparticles (NPs) scatter and absorb light in precise, designable ways, making them agile candidates for a variety of biomedical applications. When NPs are introduced to a physiological environment and interact with cells, their physicochemical properties can change as proteins adsorb on their surface and they agglomerate within intracellular endosomal vesicles. Since the plasmonic properties of metal NPs are dependent on their geometry and local environment, these physicochemical changes may alter the NPs' plasmonic properties, on which applications such as plasmonic photothermal therapy and photonic gene circuits are based. Here we systematically study and quantify how metal NPs' optical spectra change upon introduction to a cellular environment in which NPs agglomerate within endosomal vesicles. Using darkfield hyperspectral imaging, we measure changes in the peak wavelength, broadening, and distribution of 100-nm spherical gold NPs' optical spectra following introduction to human breast adenocarcinoma Sk-Br-3 cells as a function of NP exposure dose and time. On a cellular level, spectra shift up to 78.6 ± 23.5 nm after 24 h of NP exposure. Importantly, spectra broaden with time, achieving a spectral width of 105.9 ± 11.7 nm at 95% of the spectrum's maximum intensity after 24 h. On an individual intracellular NP cluster (NPC) level, spectra also show significant shifting, broadening, and heterogeneity after 24 h. Cellular transmission electron microscopy (TEM) and electromagnetic simulations of NPCs support the trends in spectral changes we measured. These quantitative data can help guide the design of metal NPs introduced to cellular environments in plasmonic NP-mediated biomedical technologies.

## Background

By virtue of their size and unique optical properties, metal nanoparticles (NPs) have enabled the development of numerous diagnostic and therapeutic technologies, such as contrast-enhanced medical imaging, plasmonic photothermal cancer therapy (PTT), and light-activated photothermal release of biomolecules [1-6]. While these technologies are extremely promising, their successful clinical adoption requires a clearer understanding of how NPs interact with the biological environment in which NPs are introduced. Already, studies have demonstrated how NPs' physicochemical properties (e.g., size and surface charge) can change when NPs are introduced into biological media and thus impact NP behavior. For example, proteins in the blood or cell culture medium can adsorb onto the NP surface, increasing NP size by as much as a factor of two [7,8], altering the surface charge [7-9], and under different conditions either preventing [10,11] or facilitating [12,13] inter-particle aggregation. These new surface and structural properties can consequently determine which cell types NPs are targeted to [14], the extent of cellular uptake [15-19], and where NPs localize within cells [7,16]. It is therefore evident that the property changes experienced by NPs in a biological environment must be well-understood so that NPs can be engineered to perform as intended in biomedical contexts.

One NP property whose changes have thus far not been well-studied in a biological environment is the metal NP optical response. When optically excited, metal NPs support a collective oscillation of surface electrons (termed ‘plasmon’), which is manifested as a strong interaction with light in terms of both absorption and scattering. The wavelength at which this optical response is strongest, termed the localized surface plasmon resonance wavelength, is very sensitive to the NP geometry and surrounding environment [20]. Both of these factors can change when NPs are internalized by cells, suggesting that the optical properties of NPs may be impacted. Specifically, during receptor-mediated endocytosis of NPs, NPs are sequestered into subcellular endosomes and trafficked to late endosomes and lysosomes, where increasing numbers of NPs accumulate and possibly agglomerate [21,22]. Intracellular agglomeration of NPs should alter the NP plasmonic spectra because from a fundamental plasmonics viewpoint, placing metal NPs in close proximity to each other can result in electromagnetic coupling of their plasmons and lead to a shift of the plasmon resonance wavelength to longer wavelengths (‘red-shift’) [23-25]. A red-shift of plasmonic NP spectra following NP agglomeration within cells has indeed been noticed in some studies. For instance, while developing anti-EGFR-conjugated gold NPs (AuNPs) as biosensors for measuring intracellular refractive index, Curry et al. observed that the scattering peaks for antibody-free AuNPs red-shifted and had a broader distribution than those for antibody-conjugated AuNPs following incubation with A431 cells [26]. Because transmission electron microscopy (TEM) showed that antibody-free AuNPs were aggregated in cells and the antibody-conjugated AuNPs were singular, Curry et al. noted that the aggregation of antibody-free AuNPs in cells contributed to a red-shift in spectra [26]. Recently, red-shifting in the spectra of gold nanorods (AuNRs) in a pellet of MDA-MB-231 cells has also been observed [27]. While these observations have suggested that the optical properties of plasmonic NPs can change in a cellular environment, such spectral changes have not yet been quantified or systematically studied, making it difficult to assess whether such changes are important to consider in the future design of plasmonic NPs for biomedical applications.

Quantitative knowledge of how the NP spectra and plasmon resonance wavelength change in a cellular environment can inform many *in vitro* and *in vivo* plasmonic NP-mediated applications. For instance, in PTT, metallic nanostructures are excited at their plasmon resonance maximum to cause localized absorption, heating, and destruction of cancer tissue. The NP geometry is designed such that the peak resonance wavelength measured in solution coincides with the emission wavelength of the laser used [4,28]. However, because the magnitude of spectral shift resulting from intracellular clustering has not been quantified, it is unknown how much of a mismatch with the laser emission wavelength can arise and if it is significant to consider. Researchers have proposed utilizing the intracellular NP agglomeration effect so that simple solid spherical AuNPs that are restricted to resonance at visible wavelengths can be excited for PTT with infrared wavelengths within the biological transparency window after agglomeration *in vivo*[29-32]. Plasmonic nanostructures are also employed in *in vitro* studies to study biological processes by using plasmon resonance energy transfer (PRET) [33]. The presence of a biomolecule with an absorption peak coinciding with the resonance peak of a plasmonic NP probe is detected by a dip in the probe's plasmon resonance spectrum [33]. Since the resonance of the NP probe must match the absorption of the molecule of interest for PRET to work, understanding the conditions under which the NP probe resonance may shift upon cellular uptake would help ensure accuracy of PRET. Finally, plasmonic NPs are being explored for multiplexing as multicolor labels for molecular imaging [34,35] or antennas for photonic gene circuits [36]. In demonstrating the potential of photonic gene circuits, Lee et al. showed that two AuNR antenna populations functionalized with small interfering RNA (siRNA) could differentially release siRNA and thus turn gene circuits ‘off’ or ‘on’ upon excitation with light at one nanorod population's resonance wavelength [36]. However, there was some release of siRNA from the nanorod antenna population that was non-resonant at the light wavelength illuminating the cells [36]. This crosstalk suggests the potential importance of developing methods to fabricate NPs with narrower resonances and considering possible spectral changes in a cellular environment when designing the plasmon resonance characteristics of NP antennas.

In this paper, we systematically investigate and quantify changes in the spectra of metal NPs *in situ* following their introduction to a cellular environment in order to inform optimized design of NPs for plasmonic NP-mediated biomedical applications. Using darkfield hyperspectral (HS) imaging, we measure optical spectra of NP clusters (NPCs) on the cell level and intracellular NPC level, and we quantify how the spectra's peak wavelength changes with time and NP exposure dose. We also characterize spectral broadening since its magnitude will impact the range of illumination wavelengths that can be used to excite NPs in a cellular environment as well as the ability for NPs to be multiplexed without spectral overlap. We find that the spectral shift is both time- and dose-dependent, accruing a maximum shift of 78.6 ± 23.5 nm between 2 and 24 h of NP incubation with cells, and that spectra broaden significantly after 24 h, reaching a width of about 105 nm at 95% of the spectrum's maximum intensity. The quantitative analysis and characterization of how the NPs' spectral peak wavelength and spectral broadness change upon interaction with cells will help guide NP design for maximal efficacy and safety in biological environments.

## Methods

### HS imaging of AuNPs in varying refractive indices

Glass slides were cleaned using piranha acid (7:3 mixture of sulfuric acid to hydrogen peroxide. *Caution: very strong acid and oxidizer!*) for 15 min followed by ethanol and DI water rinses. Slides were then immersed in 10% (3-mercaptopropyl)trimethoxysilane (MPTMS) in ethanol for at least 4 h, rinsed with ethanol, and dried with N_2_ gas. MPTMS-functionalized slides were baked at 80°C for 30 min to strengthen the glass-silane bond and kept in ethanol until use. A uniform submonolayer of NPs was immobilized on the slides by immersing slides in 20 mL of 1.12 × 10^9^ NP/mL 100-nm citrate-stabilized spherical AuNPs (Ted Pella, Redding, CA, USA) for 90 min on an orbital shaker, rinsing with ethanol and ultrapure water (Milli-Q, Millipore, Billerica, MA, USA), and drying with N_2_ gas. HS images were collected for the same NP substrate with air (*n* = 1.00), water (*n* = 1.33), or glycerol (*n* = 1.47) present between the top of the substrate and the cover slip. The same field of view (defined by the coordinates of a motorized stage) was observed under each condition, and the substrate was rinsed with Milli-Q and dried with a gentle stream of N_2_ gas before each switch into another refractive index medium.

### Mie calculations

Mie calculations were performed using a MATLAB code adapted for light scattering from concentric spheres. Extinction, scattering, and absorption coefficients were calculated based on Mie scattering coefficients. Plane-wave illumination was assumed to obtain the Mie coefficients. This assumption is justified by our use of subdiffraction-limit sized AuNPs and the fact that they were located far from the illuminating objective compared to the wavelength of visible light. Gold properties were adapted from Johnson and Christy [37].

### Characterizing spectral shift of NPs from water to CPRFM

To assess initial changes to the NP spectra upon incubation in complete phenol red-free cell culture media (CPRFM) prior to cellular uptake, extinction spectra of NPs before and after transfer to CPRFM was measured with an Agilent Cary 60 UV-vis spectrophotometer. CPRFM consisted of phenol red-free McCoy's 5A media (HyClone, Waltham, MA, USA) containing 10% v/v human off-the-clot type AB serum (PAA Laboratories, Pasching, Austria) and 1% penicillin-streptomycin (Sigma-Aldrich, St. Louis, MO, USA). Citrate-stabilized spherical AuNPs (100 nm, Ted Pella, Redding, CA, USA) were centrifuged, redispersed in CPRFM, and incubated at 37°C in 5% CO_2_ in a cell culture incubator. NPs in water (negative control) and CPRFM alone were also kept under same conditions. All spectra were collected with water as the baseline. Spectra for CPRFM alone were measured at each time point and subtracted from the corresponding spectra of NPs incubated in CPRFM.

### Measuring optical spectra for NPs introduced to cellular environment

Sk-Br-3 cells (American Type Culture Collection, Manassas, VA, USA) were cultured in McCoy's 5A media supplemented with 10% v/v human off-the-clot type AB serum and 1% penicillin-streptomycin and maintained at 37°C in a 5% CO_2_ incubator. Sk-Br-3 cells were plated on Lab-Tek II CC2 4-well chamber slides at a density of 100,000 cells/well and grown to 70% confluence. After 24 h, culture media was removed and cells were incubated with AuNPs in CPRFM for 2, 5, 10, or 24 h in an incubator at 37°C and 5% CO_2_. AuNPs in CPRFM were prepared by centrifuging citrate-stabilized AuNPs (100 nm, Ted Pella, Redding, CA, USA) at 2,000 g for 20 min, redispersing in CPRFM, sonicating, passing through a 0.22-μm polyethersulfone (PES) sterile filter (Millipore, Billerica, MA, USA), and diluting in CPRFM to final concentrations of 12, 24, 48, or 96 μg/mL. Following incubation, cells were rinsed 3x with 1X Dulbecco's phosphate-buffered saline without magnesium and calcium (PBS, Invitrogen, Life Technologies, Grand Island, NY, USA), fixed using 4% formaldehyde (15 min, BD Biosciences, San Jose, CA, USA), and rinsed again 2x with PBS. Chamber slides with cells and internalized NPs were then wetted with PBS, covered with a cover slip, sealed with nail polish, and viewed under an Olympus BX-41 upright microscope (Olympus, Center Valley, PA, USA) coupled with a CytoViva high-resolution illuminator at 40x magnification (Plan Fluorite, 0.75 NA, Olympus, Center Valley, PA, USA).

Spectral data across the sample field of view were collected using the hyperspectral imaging system connected to the CytoViva microscope (CytoViva, Auburn, AL, USA). Samples were illuminated with a quartz halogen lamp with aluminum reflector (400 to 1,000 nm) and spectral data cubes were obtained by automated movement of the sample using a X-Y motorized stage (Prior, Rockland, MA, USA) across a transmission diffraction grating spectrograph with 2.8-nm resolution (Specim, Oulu, Finland). Darkfield HS images were taken at the slide plane as well as 5 μm above the slide plane (controlled by a Prior motorized z focus drive with 0.002-μm minimum step size; see Additional file 1 for more details). HS images were analyzed using the instrument's ENVI software (ITT Visual Information Solutions, Boulder, CO, USA). Cell regions of interest (ROIs) were defined by tracing the outline of cells with the polygon ROI tool. In order to objectively define ROIs without bias toward cells that appeared to have greater uptake of NPs, all cells in each HS image were identified as long as the cell boundaries could be unambiguously identified. NPC ROIs were defined by selecting 12-pixel ellipse-shaped ROIs encompassing the image pixels for NPCs (see Additional file 1 for details). Spectral data averaged across the pixels of each ROI was extracted by the ENVI software. The spectral data was calibrated for variations in lamp intensity by dividing by the normalized lamp spectrum.

### Peak wavelength determination

Spectral peak wavelengths were objectively determined using the peak analyzer function of OriginPro 8.6 Data Analysis and Graphing Software (OriginLab, Northampton, MA, USA). Savitzky-Golay smoothing with a window size of 50 was first performed on spectral data followed with peak finding using a local maximum method with two local points. Calculated peak wavelengths were then manually checked against the plotted spectra to confirm accuracy. When spectra contained multiple peaks, the peak with higher intensity was analyzed.

Since peak wavelength could not be accurately identified in spectra that were dominated by cell scattering and had insufficient NP plasmonic spectra contributions, we excluded these spectra in our analysis. To objectively classify which spectra lacked sufficient signal for analysis, we inspected the spectra's normalized intensity at 500 nm. Spectra that had a normalized intensity at 500 nm of 0.95 or higher were deemed cell scattering-dominated and thus not included in the analysis.

### Peak broadening measurement

A custom program was created in MATLAB to smooth and calculate the peak broadening of the spectra. Spectral data was first smoothed using a Savitzky-Golay algorithm (chosen to maximally preserve the spectral shape and intensity) of degree 2. All points in the smoothed spectrum were then shifted downward by a constant value equivalent to 95% of the spectrum's maximum intensity. The smoothed and shifted spectrum's two points of zero crossing represented the span of the spectrum at 95% of the spectrum's maximum intensity. The width of the spectrum at 95% of the maximum intensity was then calculated by taking the difference of the two points of zero crossing.

### Evaluating cell viability

Cell viability after incubation with NPs was evaluated by performing a Live/Dead viability assay (Life Technologies, Grand Island, NY, USA). Following NP exposure, cells were rinsed 3x with PBS, incubated with Live/Dead reagent for 15 min at room temperature, and imaged at 20x magnification with a Zeiss Axio Observer.A1m inverted fluorescence microscope (Zeiss, Jena, Thuringia, Germany). Live cells and dead cells were visualized using filters with the following excitation and emission specifications: Ex 480 ± 20 nm/Em 535 ± 25 nm and Ex 560 ± 27.5 nm/Em 645 ± 37.5 nm (Chroma, Bellows Falls, VT, USA), respectively. Numbers of live and dead cells were counted using ImageJ (National Institutes of Health, Bethesda, MD, USA). Cell viability was determined as number of live cells/number of total cells × 100%.

### Cellular TEM

Following incubation with NPs, cells were rinsed 3x with 1X PBS, fixed with 2.5% formaldehyde/2.5% glutaraldehyde in 0.1 M sodium cacodylate buffer (Electron Microscopy Sciences, Hatfield, PA, USA) at room temperature, and kept at 4°C overnight. After fixation, the samples were washed in 0.1 M cacodylate buffer and treated with 0.1% Millipore-filtered buffered tannic acid, postfixed with 1% buffered osmium tetroxide for 30 min, and stained *en bloc* with 1% Millipore-filtered uranyl acetate. The samples were washed several times in water, then dehydrated in increasing concentrations of ethanol, infiltrated, and embedded in Spurr's low-viscosity medium. The samples were polymerized in a 60°C oven for 2 days. Ultrathin sections were cut in a Leica Ultracut microtome (Leica, Buffalo Grove, IL, USA), stained with uranyl acetate and lead citrate in a Leica EM stainer (Leica, Buffalo Grove, IL, USA), and examined in a JEM 1010 transmission electron microscope (JEOL USA, Inc., Peabody, MA, USA) at an accelerating voltage of 80 kV. Digital images were obtained using AMT Imaging System (Advanced Microscopy Techniques Corp, Woburn, MA, USA).

### Electric field simulation

The electric field was calculated using the RF module in COMSOL Multiphysics. The simulation geometry consisted of a spherical far-field scattering domain with a radius of 500 nm, a perfectly matched layer of 350-nm thickness, and clusters of AuNPs whose center coordinates are supplied in Additional file 1: Table S2. Gold properties were adapted from Johnson and Christy [37]. The refractive index of water was set to 1.33. The scattered field was calculated with a background electric field linearly polarized in the *x* direction and propagating in the *z* direction. The definition of the E-field with respect to the AuNP cluster geometry is shown in Additional file 1: Figure S11. Direct solvers were used for the simulation. Computation was performed by a workstation equipped with dual 6-core Intel Xeon X5690 3.46 GHz processors and 144 GB of RAM. The E-field was visualized in the middle cross section (xy plane) of the cluster.

### Statistical analysis

In comparisons of spectral peak wavelengths or spectral widths among different NP exposure times and doses, statistical significance was evaluated by performing one-factor analysis of variance (ANOVA) followed by a *post hoc* Tukey's HSD test for multiple comparisons. *P* values less than 0.01 were considered statistically significant.

## Results and discussion

For this study, we chose 100-nm spherical AuNPs as our model plasmonic NP because their plasmonic spectra are well-characterized, they have a large scattering cross section, and plasmonic nanostructure-based biomedical applications commonly utilize NPs in this size regime [1,38,39]. In addition, the extinction and scattering spectral profiles of 100-nm AuNPs are similar (Additional file 1: Figure S1), enabling comparison between darkfield HS imaging scattering spectra and UV-vis spectroscopy extinction spectra. We employed darkfield HS imaging to measure NPC spectra in a cellular context since it allows for spectral image cubes to be captured in which full reflectance spectra can be extracted from each pixel of the image. Darkfield HS imaging has been previously utilized successfully with antibody-conjugated NP probes to answer fundamental biological questions such as the density and spatial distribution of cell surface receptors [26,40-45].

### Darkfield HS imaging measures spectral data across sample with sensitivity to spectral changes

**Figure 1:**
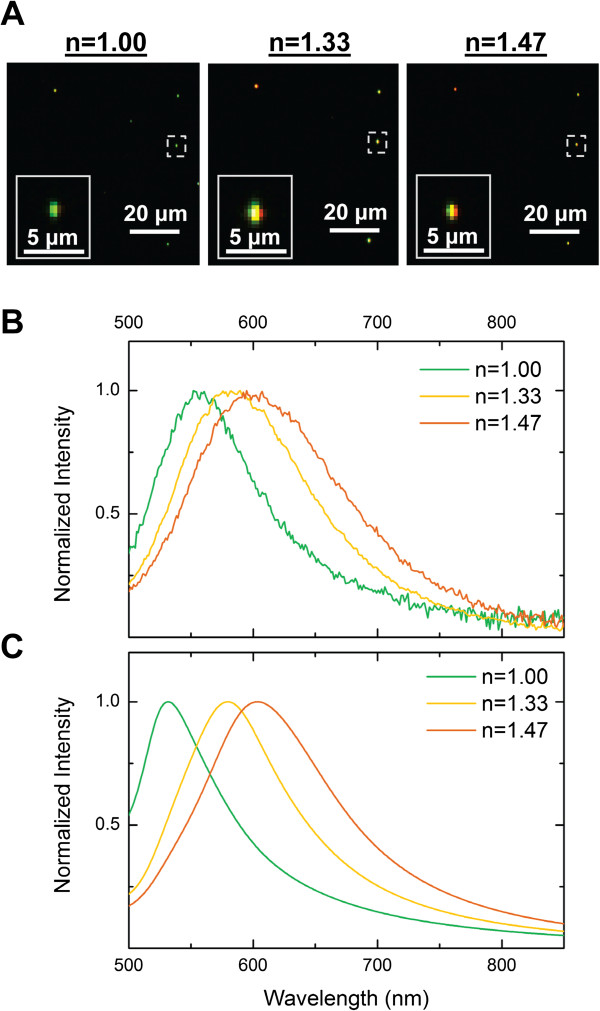
**Darkfield hyperspectral (HS) data of AuNPs in various refractive index media to confirm spectral sensitivity. (A)** HS images (taken at 40x magnification) of 100-nm AuNPs immobilized on a glass slide and imaged in air (*n* = 1.00), water (*n* = 1.33), or glycerol (*n* = 1.47), respectively. Inset shows a selected NP (or NP cluster) at higher magnification. **(B)** Scattering spectra extracted from HS image for a specific NP or NP cluster indicated by a dotted rectangle in part A. **(C)** Theoretical scattering spectra calculated based on Mie scattering theory code for 100-nm AuNPs in a homogeneous dielectric environment.

**Figure 2:**
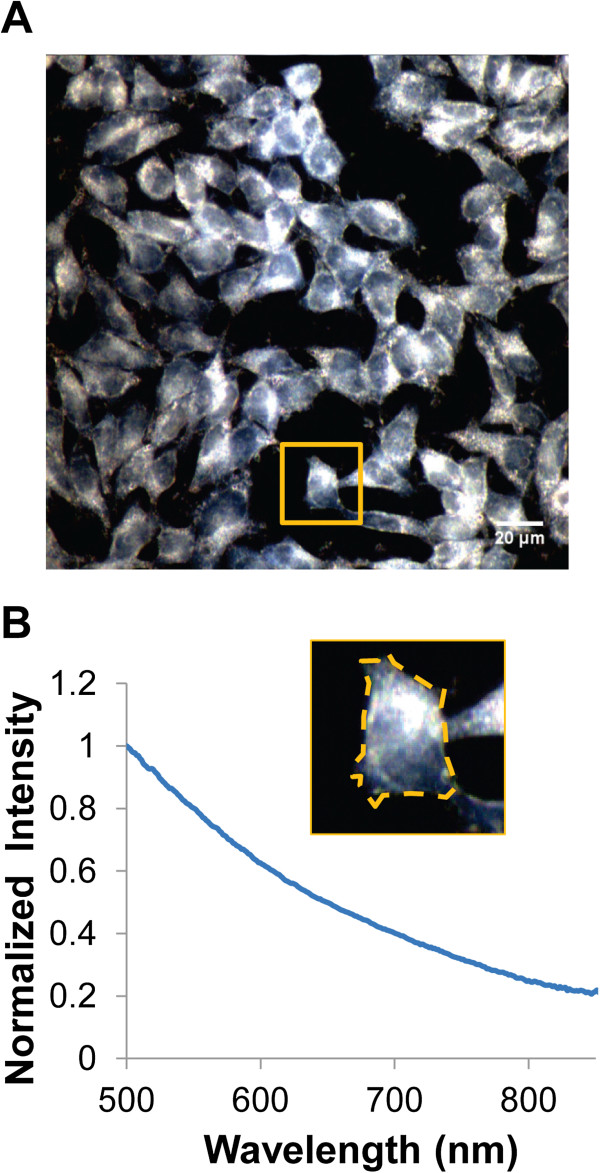
**Darkfield HS image and spectrum of Sk-Br-3 cells alone agrees with characteristic cell scattering spectra. (A)** HS image of Sk-Br-3 cells alone. **(B)** Normalized scattering spectrum of representative cell from part A. Inset shows the cell region of interest (ROI) over which the spectrum was collected.

Before quantifying changes to NP spectra in a cellular environment, we first verified that accurate spectra could be obtained and spectral shifts could be detected with sufficient sensitivity using a commercial CytoViva HS Imaging System (CytoViva, Auburn, AL, USA). A dilute solution of 100-nm spherical AuNPs was immobilized onto a glass slide and imaged using the CytoViva HS Imaging System. The same field of view of the AuNP substrate was imaged in air (*n* = 1.00), water (*n* = 1.33), and glycerol (*n* = 1.47) for validation across a range of refractive indices (RIs), including those found within a cell (*n* = approximately 1.35 to 1.38) [46,47]. In air, water, and glycerol, the NPs scattered light with a green, yellow, and yellow-orange color, respectively (Figure 1A). This was expected since the NP plasmon resonance is dependent on the RI of its local environment [20]. Figure 1B shows the measured scattering spectra of a NP selected from the HS image in air, water, and glycerol, illustrating the shift in the plasmon resonance to longer wavelengths with an increase in the RI of the surrounding medium. This shift agrees well with that predicted by Mie scattering theory for NPs well-dispersed in a homogeneous dielectric environment (Figure 1C). We further validated our system by analyzing the scattering spectrum collected from HS images of cells without NPs. The spectrum agreed with the characteristic scattering response of cells, which monotonically decreases with longer wavelengths of light (Figure 2). Together, these results suggest that the CytoViva HS Imaging System can be utilized in this study to obtain accurate cell spectra and plasmonic NP spectra with enough sensitivity to detect even shifts due to changes of RI.

### Characterizing spectral shift in media prior to cell uptake

**Figure 3:**
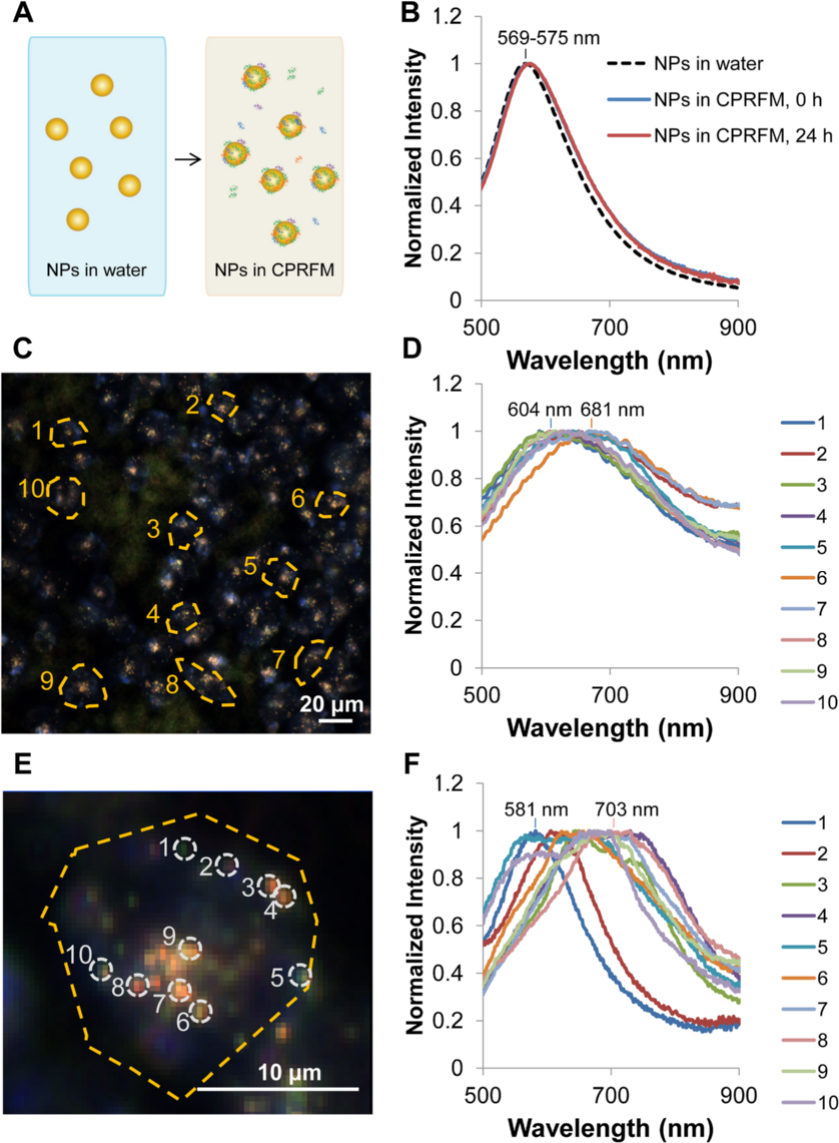
**Optical spectra for AuNPs before and after introduction to cellular environment. (A)** Schematic illustration of the transfer of NPs from water to complete phenol red-free cell culture medium (CPRFM) which results in protein corona formation and possible spectral shift prior to cellular uptake. **(B)** UV-vis extinction spectra of NPs before transfer to CPRFM, immediately after transfer to CPRFM, and after 24 h of incubation in CPRFM, showing minimal spectral shift in CPRFM prior to cellular uptake. **(C)** Representative darkfield HS image of Sk-Br-3 cells exposed to 24 μg/mL AuNPs for 24 h and **(D)** corresponding spectra extracted from the 10 representative cell ROIs denoted in part C. **(E)** Darkfield HS image of part C's cell ROI 2 with 10 representative ROIs drawn around NP clusters (NPCs). **(F)** Optical scattering spectra measured from the 10 NPC ROIs defined in part E. Number labels above spectra in parts D and F indicate range (minimum and maximum) of peak wavelengths. For clarity, only 10 representative ROIs are shown in parts C and E.

In order to quantify the changes in NP spectra that occur when NPs are introduced to a cellular environment, we transferred citrate-stabilized 100-nm AuNPs into complete cell culture media and incubated them with cells. When NPs are transferred to complete cell culture media, a protein corona can form on their surface, and they may agglomerate; thus, even before cellular uptake, it is possible for there to be some shift in the NP spectra. We characterized the shift in NP spectra due to the transfer of NPs to cell culture media before cellular uptake by using UV-vis extinction spectroscopy (Figure 3A,B). The AuNPs originally had a resonance peak wavelength of 569 nm in water. After transferring the NPs into complete phenol red-free cell culture media containing 10% human serum (CPRFM), the spectra red-shifted 5 nm to a peak wavelength of 574 nm. Even after 24 h of incubation in CPRFM, NP spectra cumulatively shifted no more than 6 nm to a peak wavelength of 575 nm (Figures 3A,B, and Additional file 1: Figure S2). These values agree well with previous reports of a 4- to 6-nm shift by 10- to 80-nm AuNPs after 24 h in cell culture media [48,49] and represent the shift in NP spectra possible before cellular uptake.

### Quantifying shift in spectral peak wavelength of NPs upon introduction to cellular environment

To quantify changes in the optical spectra of AuNPs following cellular uptake, we exposed human breast adenocarcinoma Sk-Br-3 cells to NPs in CPRFM. We chose to use the Sk-Br-3 cell line because it is commonly used in studies to demonstrate PTT [4,28]. After incubation with NPs for up to 24 h, cells were rinsed thoroughly, fixed, and characterized by darkfield HS imaging. We measured the optical spectra from HS images by defining ROIs around either individual cells (‘cell ROI’) or around each NPC within the cells (‘NPC ROI’). Figure 3C,E shows representative HS images that illustrate how cell ROIs and NPC ROIs within those cell ROIs were defined. Spectra integrated across representative ROIs are shown in Figure 3D,F. At this exposure dose and time, both cell ROI and NPC ROI spectra showed red-shifting with respect to the original NP spectra in water or CPRFM that was greater than could be explained by simply the minor difference in RI of water (*n* = 1.33) and the surrounding fluid within cells (*n* = 1.35 ~ 1.38) [46,47] (Figure 3B,D,F; Figure 1B).

Using the cell and NPC ROI analysis approaches, we systematically quantified the spectral changes experienced by NPs in a cellular environment as a function of NP exposure duration and dose - two important parameters in administering NPs for biomedical applications. HS images were taken after cells were exposed to 12, 24, 48, or 96 μg/mL of NPs in CPRFM for 2, 5, 10, or 24 h to study the extent to which spectra change and how they change as a function of exposure duration and dose. These NP exposure doses were chosen based on the doses previously utilized in *in vitro* and *in vivo* plasmonic NP-based biomedical studies [38,50] and the NP uptake capacity of cells on a NPs-per-cell basis [22,30,51,52].

**Figure 4:**
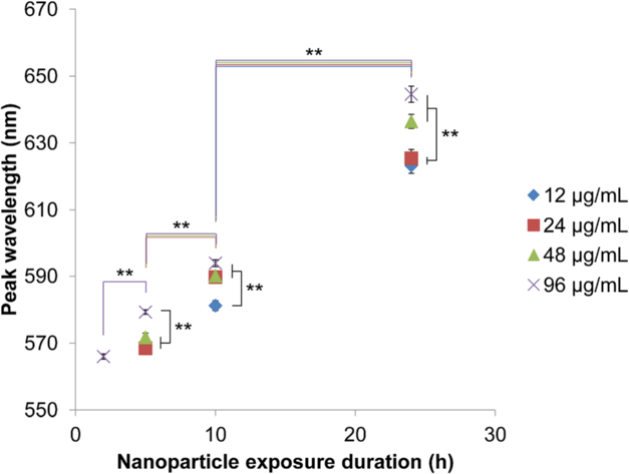
**Peak wavelengths for cell ROI spectra following NP exposure at various doses and durations.** Cells were exposed to 12, 24, 48, or 96 μg/mL AuNPs for 2, 5, 10, or 24 h. Peak wavelength increased (spectra red-shifted) with increasing NP exposure time and dose. To note, at 2 h, only cells exposed to the 96 μg/mL dose of AuNPs exhibited sufficient NP signal so that a peak wavelength could be determined. At all lower exposure doses (12, 24, 48 μg/mL) at 2 h, the measured spectra was dominated by cell scattering so peak wavelengths could not be calculated and are not shown. Error bars represent the standard error of the mean. Spectra were measured from 63 to 125 cells in each condition, but only spectra with sufficient NP signal were included in the analysis (see ‘Methods’ section and Additional file 1 for details). **p < 0.01. Color of the horizontal line underneath the **symbol indicates the exposure dose for which the comparison is made.

**Figure 5:**
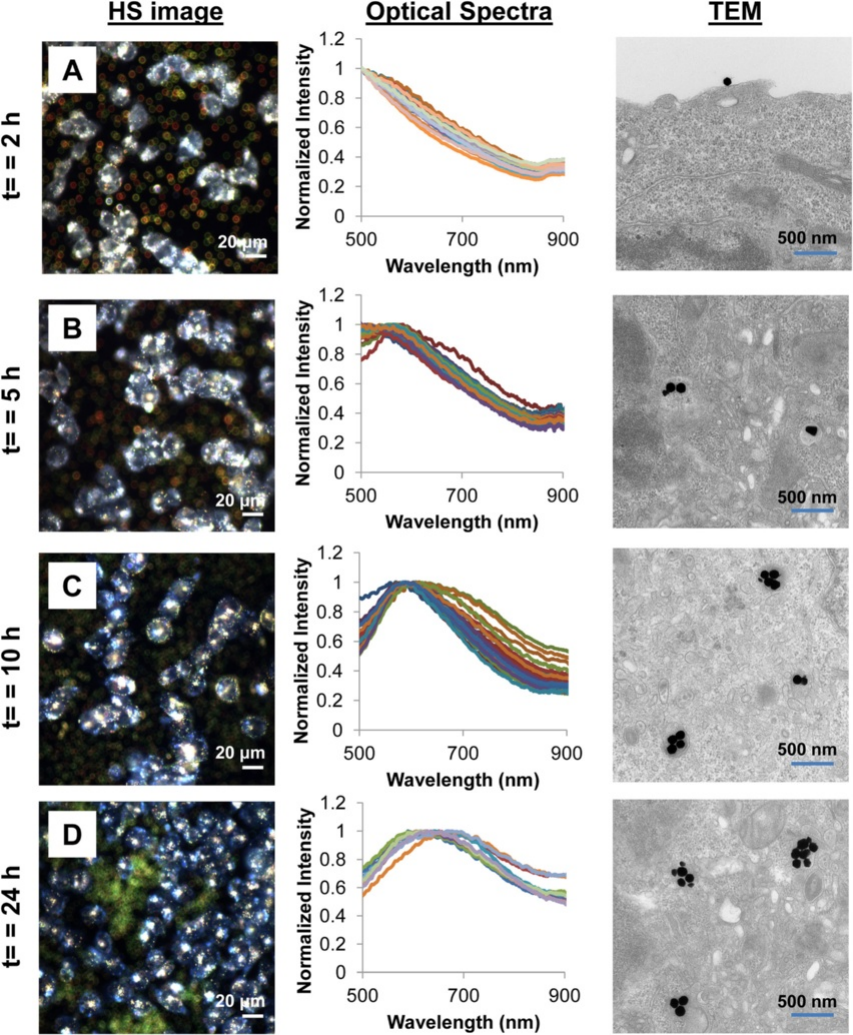
**Representative HS images, corresponding spectra, and TEM images through time following NP introduction to cells.** Cells were exposed to NPs at a dose of 24 μg/mL for **(A)** 2-, **(B)** 5-, **(C)** 10-, and **(D)** 24-h incubation. The continual increase in NP cluster size within cells with increased exposure time seen in cellular TEM images suggests that intracellular agglomeration of NPs contributes to the measured spectral shifts. Spectra are normalized to 1 for ease of comparison. HS images were taken at 5 μm above the slide plane to capture spectral contributions from intracellular NPs (see ‘Methods’ section and Additional file 1).

Spectral analysis based on cell ROIs showed that spectra red-shifted with increasing exposure time and NP exposure dose (Figure 4). Figure 5 shows representative HS images, corresponding spectra, and cellular TEM images to illustrate the progression of changes observed in HS images, spectra, and cellular TEM images after 2, 5, 10, and 24 h of NP exposure at the 24 μg/mL dose. Initially after only 2 h of NP exposure, few NPs were observed to be associated with cells in HS images (Figure 5A). Cellular TEM images indeed confirmed the presence of only 0 or 1 NP on the cell surface or interior of cell slices at *t* = 2 h (Figure 5A). Spectra extracted from cell ROIs in the HS images showed monotonically decreasing optical spectra, suggesting that cell scattering dominated over the signal from the few NPs internalized by cells at this early time point (Figure 5A). Interestingly, at this early time point, spectra for the other low exposure doses (12 and 48 μg/mL) were also dominated by cell scattering. Only the highest exposure dose (96 μg/mL) of NPs resulted in high enough NP uptake at 2 h to provide enough NP signal in the spectra and enable the spectral peak wavelength to be determined (Figure 4).

After 5 h of exposure to NPs, a greater presence of NPs was observed in association with some cells (Figure 5B). Cellular TEM showed there were more NP-containing vesicles in cells at this time than at *t* = 2 h; however, each vesicle in the cell slice only contained 1 to 3 NPs and most commonly only contained 1 NP (Figure 5B and Additional file 1: Figure S3B. *Note*: *Number of NPs in vesicles from cell slices does not represent the total number of NPs in 3D vesicles, but are provided to help in following relative trends*). Spectra extracted from cell ROIs in the HS images taken at this time point were either still dominated by cell scattering or showed a peak wavelength of 568.5 ± 7.7 nm. After 10 h of NP exposure (Figure 5C), HS images showed that the majority of cells contained a large number of NPCs. Spectra from the HS images appeared broader and shifted to longer wavelengths, with a mean peak wavelength of 589.7 ± 10.3 nm. This corresponded well to the increase in the number of NPs per NPC and proportion of cells containing multiple NPCs (most visualized cell slices contained 3 to 5 NPCs) observed by cellular TEM (Figure 5C and Additional file 1: Figure S3C). Following 24 h of NP exposure (Figure 5D), nearly all cells in the HS images contained areas with large numbers of NPCs, and the mean spectral peak wavelength reached 625.4 ± 27.3 nm.

Overall, cell ROI spectral analysis showed that a mean spectral shift of 54 to 79 nm was possible over 24 h at the exposure doses studied here (Figure 4). At the lowest dose of 12 μg/mL, cell ROI spectra showed peak wavelengths of 569.7 ± 7.8 and 623.2 ± 24.8 nm after 5- and 24-h NP exposure, respectively, for a total shift of 53.5 ± 26.0 nm (Figure 4). At the highest NP exposure dose (96 μg/mL) tested, cell ROI spectra showed peak wavelengths of 566.0 ± 4.6 and 644.6 ± 23.0 nm after 2 and 24 h of NP exposure, respectively, for a total shift of approximately 78.6 ± 23.5 nm (Figure 4). At the exposure doses used in this study, cells remained >97% viable based on a Live/Dead viability assay, and therefore, the measured spectral shifts represent shifts that are possible in normal functioning cells with an intact cell membrane (Additional file 1: Figure S4). Greater spectral shifts were experienced when higher NP exposure doses were used: After 5-h incubation, cells exposed to 96 μg/mL NPs had a significantly greater peak wavelength than those exposed to 12, 24, or 48 μg/mL NPs (Figure 4). At 10 h, the 24, 48, and 96 μg/mL NP exposure doses had significantly greater spectral peak wavelengths than those of the 12 μg/mL exposure dose. Finally after 24-h exposure, the 48 and 96 μg/mL doses achieved significantly greater spectral peak wavelength than cells exposed to the 12 and 24 μg/mL doses (Figure 4). One of the reasons for this finding may be that higher NP doses show faster uptake by cells [22], which results in larger accumulation of NPs. Additionally, NPs that are internalized early have more time to be transported to larger intracellular vesicles, thus forming large NPCs.

While TEM showed a continual increase in NP cluster size inside cells with increasing exposure time (Figure 5 and Additional file 1: Figure S3), indicating that agglomeration of NPs within cellular endosomes contributes to the spectral shift we measured, we cannot rule out the possibility that some NPs may remain adsorbed to the cell surface after rinsing. Therefore, the spectra we report here represent the total contribution from both internalized NPs as well as any NPs still on the cell exterior. This situation is actually similar to what happens when metal NPs are introduced *in vitro* or *in vivo*, where both internalized and cell surface-bound NPs will contribute a plasmonic response upon illumination [53].

### After 24 h, cell ROI spectra are heterogeneous

**Figure 6:**
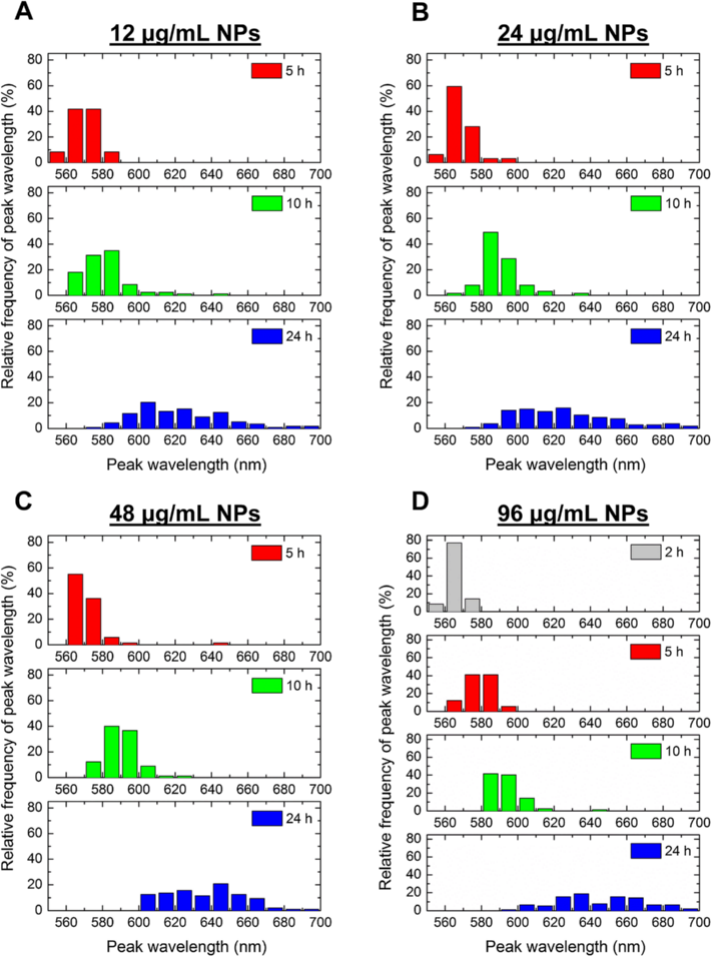
**Distribution of spectral peak wavelengths measured from different cells of same population.** Cells were exposed to **(A)** 12, **(B)** 24, **(C)** 48, and **(D)** 96 μg/mL of AuNPs for 2, 5, 10, or 24 h. Cell ROI spectral peak wavelengths among different cells increase in diversity with time. To note, at 2 h, only cells exposed to the 96 μg/mL dose of AuNPs **(D)** exhibited optical spectra for which a peak wavelength could be determined. At all lower exposure doses **(A, B, C)** at 2 h, the measured spectra was dominated by cell scattering.

While Figure 4 shows the mean peak wavelengths exhibited by cells following NP exposure, the distribution of peak wavelengths across cells in a cell population is important for determining how homogenous of an optical response can be achieved from cell to cell in a cell population. Figure 6 depicts the distribution of spectral peak wavelengths exhibited by the various cells of a cell population and provides a sense of the range and degree of heterogeneity of spectral peak wavelengths among cells. For all exposure doses, cells incubated with NPs for 2, 5, or 10 h showed a relatively narrow distribution of spectral peak wavelengths, but after 24 h of NP incubation, substantial heterogeneity in spectral peak wavelengths exhibited by different cells was observed (Figure 6). For example, cells exposed to 12 μg/mL of NPs for 24 h showed spectra ranging from 573 to 696 nm in peak wavelength (Figure 6). This large spread of peak wavelengths may be attributed to cells each being in different stages of the cell cycle, with some cells just starting to internalize NPs and other cells already having internalized and processed NPs from early endosomes to late lysosomes for a longer time. Diversity in the number of NPs per vesicle and number of vesicles per cell has recently been reported [54], and we also observe this variability in NP distribution among cells in cellular TEM images (Additional file 1: Figures S3, S5). Additionally, heterogeneity in cellular uptake and agglomeration of NPs has previously been reported for scavenger receptor-mediated endocytosis of silver NPs by J774A.1 macrophage cells [45], similar to what we observed in our general receptor-mediated endocytosis experimental setup here. In summary, we observe that a cell population exposed to NPs will exhibit an increasing diversity in optical spectra with time. The increased heterogeneity of cell ROI spectra with time suggests that it will be important to consider timing when exciting NPs for applications based on a cellular level optical response.

### Cell ROI spectra broaden significantly between 10- and 24-h incubation

**Figure 7:**
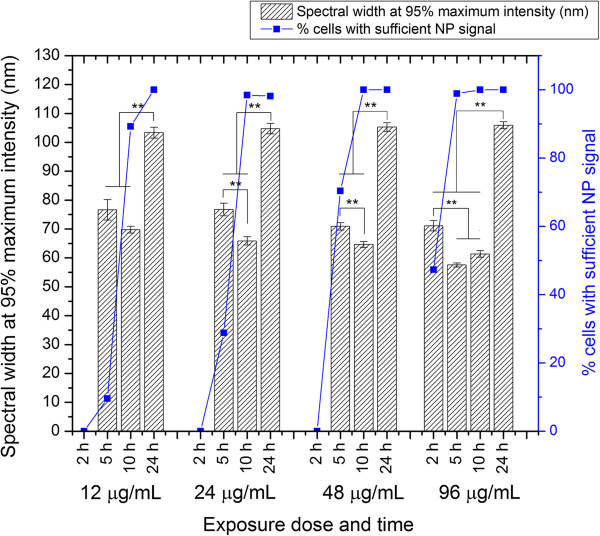
**Broadness of cell ROI spectra.** Cells were exposed to 12, 24, 48, or 96 μg/mL of AuNPs for 2, 5, 10, or 24 h. Spectral broadness was defined as the spectral width at 95% of maximum intensity (‘spectral width’). Spectral width increased significantly after 24 h of exposure (** denotes *p* < 0.01). Shown are the mean and standard error of the mean. For each condition, spectra were analyzed from 63 to 125 cells. The percent of cells exhibiting sufficient NP signal for analysis is also plotted. At low exposure dose and duration, only a small proportion of cells in the cell population displayed sufficient NP signal since most cells had not yet internalized a substantial number of NPs at this time. Spectral width was only analyzed for cells containing sufficient NP signal (not dominated by cell scattering); see Additional file 1 for details. Thus, at low exposure doses and durations, the spectral width measurements reflect only the small subset of cells that had internalized NPs and thus had sufficient NP signal to be analyzed.

We next examined the broadness of the cell ROI spectra since the broadness of the resulting spectra of NPs in cells will impact the wavelength range in which the NPs can be excited. We defined broadness by measuring the spectral width at 95% of the maximum intensity (‘spectral width’). The mean spectral width ranged between 57.6 ± 6.4 and 76.8 ± 12.5 nm for all NP exposure doses after 5- and 10-h incubation, respectively, but spectra experienced further broadening (along with red-shifting) after 24 h of cellular exposure to NPs (Figure 7). After 24 h of NP exposure, cell ROI spectra showed spectral widths ranging from 103.4 ± 20.3 to 105.9 ± 11.7 nm for the different exposure doses. Spectral width increased significantly between early time points and 24 h of NP exposure (Figure 7, *p* < 0.01). Interestingly, for cells exposed to 24 and 48 μg/mL exposure doses, the mean spectral width decreased between 5 and 10 h. This may be because at 5-h exposure, only a subset of cells in the cell population had sufficient NP signal for spectral analysis (Figure 7, see ‘Methods’ section for how cells were objectively classified as having sufficient NP signal for analysis), and the mean spectral width thus only reflected this specialized subpopulation. The relative broadness of the cell ROI spectra suggests that a wider range of wavelengths may be used to excite the NPs and still achieve an optical scattering response with intensity between 95% and 100% of that achieved at the peak wavelength.

We attribute the broadening of spectra at *t* = 24 h mainly to the longer period of time provided for cells to accumulate NPs and process them into various endosomes and lysosomes, which contributes to larger degrees of heterogeneities in the NPC sizes and geometrical configurations in the cell ROI. The simultaneous presence of newly endocytosed NPs of modest NPC sizes and larger NPCs accumulated from an earlier time creates a range of NPCs confirmed by cellular TEM images (Additional file 1: Figure S3). Averaging of the disparate spectra from the various NPC configurations inside the cell plays a major role in increasing the broadening of cell ROI spectra. The broadening is also contributed by the complex spectral characteristics of individual large NPCs, as will be discussed next.

### NPC ROI spectra represent optical response from individual NPCs

While cell ROI spectra reflect the overall optical response from cells containing NPCs, they do not represent local spectral properties attributed by specific NPCs. We defined ROIs around NPCs and hypothesized that their corresponding spectra could provide a closer measure of the plasmonic properties of specific NPCs within cells. For optically triggered subcellular applications of AuNPs, this type of spectral information can be important for knowing how NPCs will optically respond, and if spectral overlap is of possible concern (by assessing broadness of the NPC ROI spectra). We focused our analysis on the 24 μg/mL exposure dose at *t* = 2, 5, 10, and 24 h. For each time point, we analyzed at least 10 cells for which we previously analyzed cell ROI spectra. Within these cells, we analyzed spectra for all present NPC ROIs (*n* = 56 to 180 NPC ROIs per condition).

Like the cell ROI spectra, NPC ROI spectra showed an increasing red-shift of the peak spectral wavelength and spectral broadening over time. At all exposure times, NPC ROI spectra had greater mean peak wavelengths than those measured from cell ROIs (Additional file 1: Figure S6). We attribute the greater mean peak wavelength of NPC ROI spectra to the decreased contribution of cell scattering in NPC ROI spectra, which are only defined around individual NPCs. In other words, because cell ROI spectra are measured by integrating across a whole cell, cell ROI spectra may experience a dampened red-shift caused by the cell scattering contributions that have higher intensities at lower wavelengths (Figure 2).

**Figure 8:**
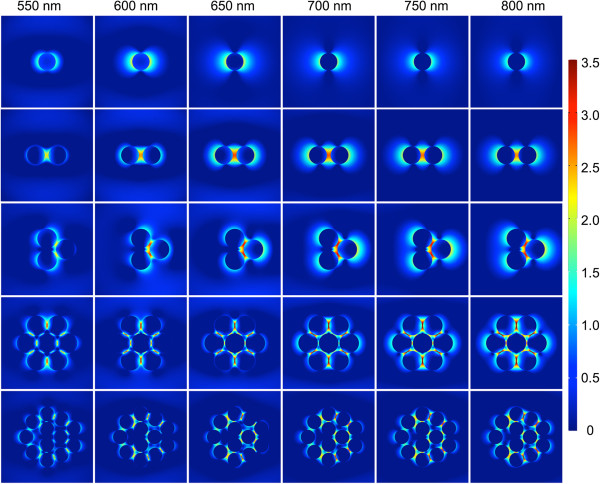
**Electromagnetic simulations illustrating the complex spectral properties of large nanoparticle clusters.** Normalized electric field plot in log scale, i.e., log(normE), in the middle plane of clusters composed of 1, 2, 3, 7, and 12 spherical 3D AuNPs (diameter 100 nm). The distance between adjacent AuNPs is between 5 and 20 nm. Inter-particle distances can be determined from the specific coordinates of the AuNPs, listed in Additional file 1: Table S2. The surrounding medium is water. Polarization of the excitation is in the horizontal direction.

Spectral broadening was also observed from NPC ROIs. Spectral width ranged from 48.7 ± 14.9 nm after 2-h NP exposure to 64.9 ± 24.5 nm after 24-h exposure (Additional file 1: Figure S7B). After 24 h of cellular exposure to NPs, NPC ROI spectra were significantly broader than after 2, 5, or 10 h of exposure (Additional file 1: Figure S7B, *p* < 0.01). We attribute this broadening to the spectral properties of the larger NPCs seen in TEM at *t* = 24 h (Additional file 1: Figure S3), as large NPCs exhibit more complex spectral properties than monomers and dimers due to the excitation of higher-order modes [55]. Inter-particle coupling, which contributes to the overall optical response of a NPC, can be wavelength-dependent; for larger NPCs, this coupling can be manifested at a broad range of wavelengths red-shifted from the plasmonic resonance of individual AuNPs. Although it is difficult to recapitulate the NPC configurations found in cells exactly with simulations, we demonstrate the complex nature of inter-particle coupling using a full-wave electromagnetic simulation program COMSOL Multiphysics. We created five NPC configurations resembling the most frequently observed geometries in cellular TEM images (Additional file 1: Figures S3, S5) and plotted the electric field between AuNPs in the logarithmic scale at six excitation wavelengths from 550 to 800 nm (Figure 8). For a 100-nm single AuNP in water, the peak resonance wavelength was between 550 and 600 nm (first row of Figure 8), consistent with the Mie calculation of 580 nm. For a dimer structure with a 20-nm inter-particle gap, the resonance peak (i.e. strongest inter-particle coupling) is red-shifted to between 650 and 700 nm (second row of Figure 8). The larger 3-, 7-, and 12-NP clusters exhibit various degrees of strong electric fields around/within the NPCs at wavelengths longer than 700 nm (third, fourth, and fifth row of Figure 8), illustrating how large NPCs exhibit more complex spectral properties than monomers and dimers. Spectral broadening can also arise from other factors not characterized by simulations in Figure 8. Major among these is the polarization of the excitation light. Excitation polarized along different geometrical axes of asymmetrical NPCs may generate distinct spectral shapes. Because our experiments were performed with unpolarized light, the measured spectra represent the averaging of spectra from different polarizations, and thus, broadening of the spectral shape. Taken together, these effects support our observation that even on a NPC level, spectra have broadened significantly through time in a cellular environment.

## Conclusions

In conclusion, we have quantified changes in the peak wavelength, broadness, and distribution of AuNP spectra after NPs are introduced to a cellular environment. As creative, new biomedical applications utilizing the optical properties of plasmonic NPs continue to be developed, it is important to understand how the NP spectra change in a physiological environment. We have shown that when 100-nm AuNPs are exposed to Sk-Br-3 breast adenocarcinoma cells, an average shift of approximately 79 nm in the optical spectra can be achieved. As the incubation time increases and cells have a chance to process NPs into larger endosomal vesicles containing multiple NPs (as indicated by TEM), the spectra continue to shift with time. At shorter incubation times following NP exposure, smaller shifts were observed, suggesting that the time at which the NPs are optically excited is important to consider. For 100-nm AuNPs, the NPC ROI spectral peak wavelength stays within 30 nm of its initial peak wavelength in the first 2 to 5 h of incubation but shifts significantly between 10 and 24 h.

The spectral shift is also sensitive to the NP exposure dose. Since NPs are internalized more quickly with higher doses, a greater shift is experienced. Although we measured a pronounced spectral shift with increasing time and exposure dose, we found that the cellular level spectra can also broaden significantly after 24 h of incubation such that at least 95% of the maximum optical response can be achieved with illumination wavelengths within a 105-nm region around the spectral peak wavelength. Given this magnitude of spectral broadening, it may not be as necessary to precisely account for where the peak wavelength is located when designing NP systems for applications based on a cellular level optical response since a similar intensity of optical response may be achieved using a wide range of wavelengths. However, as methods for fabricating NPs with narrower resonances are established, the broadening and shifting of NP spectra in cells may merit consideration in the design of NP systems for multiplexing applications where discrete NPs are excited.

The magnitude of spectral shifting and broadening experienced by NPs will likely vary for NPs with different size and surface conjugation since these properties influence protein corona composition, cellular uptake speed, and NP clustering dynamics within the cell. The HS imaging and analysis approaches utilized here may be applied to study how the optical spectra of other nanostructures with varying morphologies are affected by a cellular environment. Such studies can help build a foundation for understanding and predicting how designable NP parameters such as size and surface functionalization relate to the amount of shift and broadening experienced.

To address potential agglomeration-associated red-shifting and broadening of NPs, plasmonic NPs may be designed to aggregate less or be separated a greater distance from other NPs when internalized within cells. For example, NPs may be designed with a thick surface coating [56] that minimizes coupling of resonances within endosomes and preserves the originally designed optical NP spectra. Alternatively, further characterization of the agglomeration and resulting changes to spectra for NPs under a variety of physiological conditions can enable these agglomeration-associated optical changes to be factored into the design of NPs for biomedical applications.

## Abbreviations

AuNPs: gold nanoparticles; AuNRs: gold nanorods; CPRFM: complete phenol red-free cell culture media; HS: hyperspectral; MPTMS: (3-mercaptopropyl)trimethoxysilane; NPs: nanoparticles; NPC: nanoparticle cluster; PBS: phosphate-buffered saline; PRET: plasmon resonance energy transfer; PTT: photothermal therapy; RI: refractive index; ROI: region of interest; TEM: transmission electron microscopy.

## Competing interests

The authors declare that they have no competing interests.

## Authors’ contributions

AC designed and conducted the nanoparticle-cell and hyperspectral imaging experiments, developed spectral analysis approaches, performed analyses, interpreted results, and drafted the manuscript. YH designed and performed the electromagnetic simulations, interpreted results, and co-drafted the manuscript. AC, JY, and RD also participated in the design and interpretation of the electromagnetic simulations. MJ helped perform the nanoparticle-cell experiments, participated in the study design and interpretation of results, and assisted in the drafting of the manuscript. AL, JY, and RL participated in experimental design, interpretation of results, and revision of the manuscript. RD guided the study, participated in the experimental design and interpretation of results, and assisted in the drafting of the manuscript. All authors read and approved the final manuscript.

## Supplementary Material

Additional file 1 **Supplementary data.** A document containing eleven supplementary figures and two supplementary tables. Optical extinction, scattering, and absorption of 100-nm AuNPs; additional cellular TEM images; cell viability results; NP cluster ROI spectral measurement results; details on HS imaging and analysis rationale; and electric field calculation setup. Click here for file
