# Supplementary material for: Quantifying spectral changes experienced by plasmonic nanoparticles in a cellular environment to inform biomedical nanoparticle design
Source: Nanoscale Res Lett. 2014 Aug 31;9(1):454. doi: 10.1186/1556-276X-9-454 (PMC4164329; doi:10.1186/1556-276X-9-454)
Supplement: Additional file 1 — Supplementary data. A document containing eleven supplementary figures and two supplementary tables. Optical extinction, scattering, and absorption of 100-nm AuNPs; additional cellular TEM images; cell viability results; NP cluster ROI spectral measurement results; details on HS imaging and analysis rationale; and electric field calculation setup. [file 1556-276X-9-454-S1.pdf]

Additional File 1: Supplementary Data

Quantifying Spectral Changes Experienced by Plasmonic Nanoparticles in a Cellular Environment to Inform Biomedical Nanoparticle Design

Allen L. Chen,[1] Ying S. Hu,[2] Meredith A. Jackson,[1] Adam Y. Lin,[1] Joseph K. Young,[3]

*Robert J. Langsner,[1] Rebekah A. Drezek[1,3,]**

[1]Department of Bioengineering, Rice University, Houston, TX 77005

[2]Waitt Advanced Biophotonics Center, Salk Institute for Biological Studies, La Jolla, CA 92037

[3]Department of Electrical and Computer Engineering, Rice University, Houston, TX 77005

Contents

1. Optical extinction, scattering, and absorption of 100 nm AuNPs 2
2. Characterizing nanoparticle spectral shift in media before cell internalization 3
3. Additional cellular TEM images: *varying incubation time* 4
4. Cell viability results 8
5. Additional cellular TEM images: *varying exposure dose* 9
6. Nanoparticle cluster ROI spectral peak wavelength and broadness measurements 12
7. Additional darkfield HS imaging methods and supporting rationale 14
8. HS image analysis and sample sizes 17
9. Electric field calculation setup 18
10. References 19

1. Optical extinction, scattering, and absorption of 100 nm AuNPs

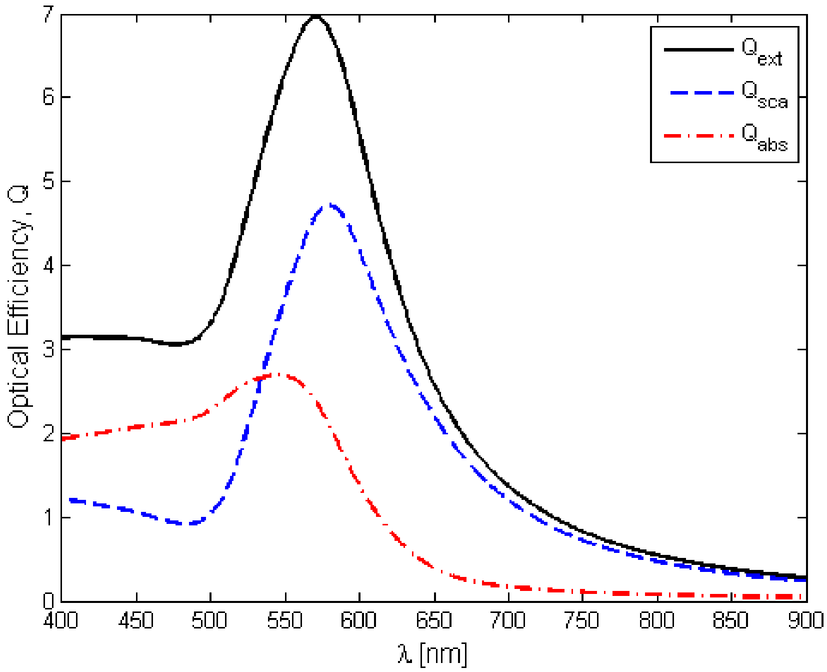

Figure S1 Optical efficiency of extinction, scattering, and absorption calculated for 100 nm AuNPs in water (n=1.33) using Mie scattering theory. AuNPs with a 100 nm diameter have similar extinction and scattering profiles.

2. Characterizing nanoparticle spectral shift in media before cell internalization

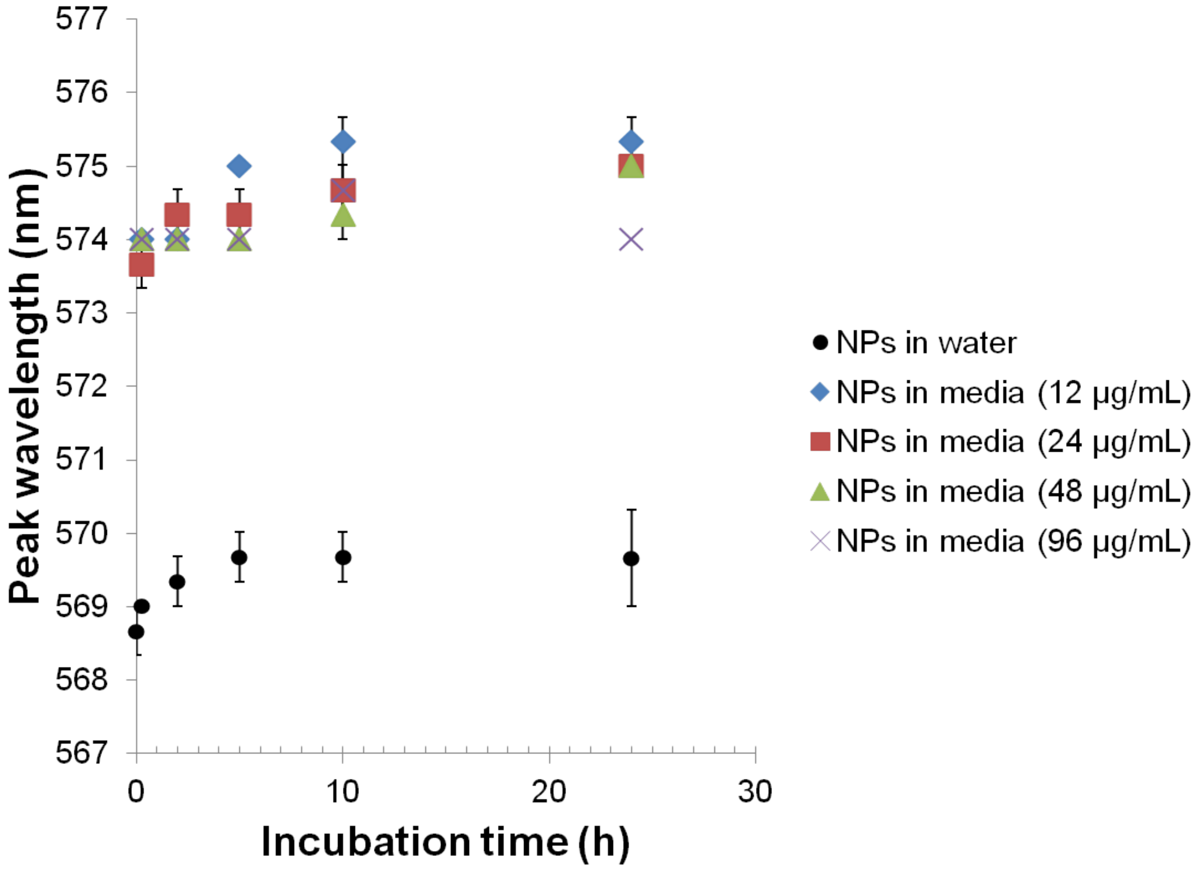

Figure S2 Peak wavelength of NP extinction spectra before and after transferring NPs to complete cell culture medium (containing 10% human serum) at 37°C for 24 h. Error bars represent standard error of the mean for n=3. After transferring NPs to cell culture medium, NP spectra red-shifted approximately 5-6 nm due to culture medium effects such as protein corona formation on NP surface. During the same period, NPs in water shifted no more than 1 nm.

3. Additional cellular TEM images: *varying incubation time*

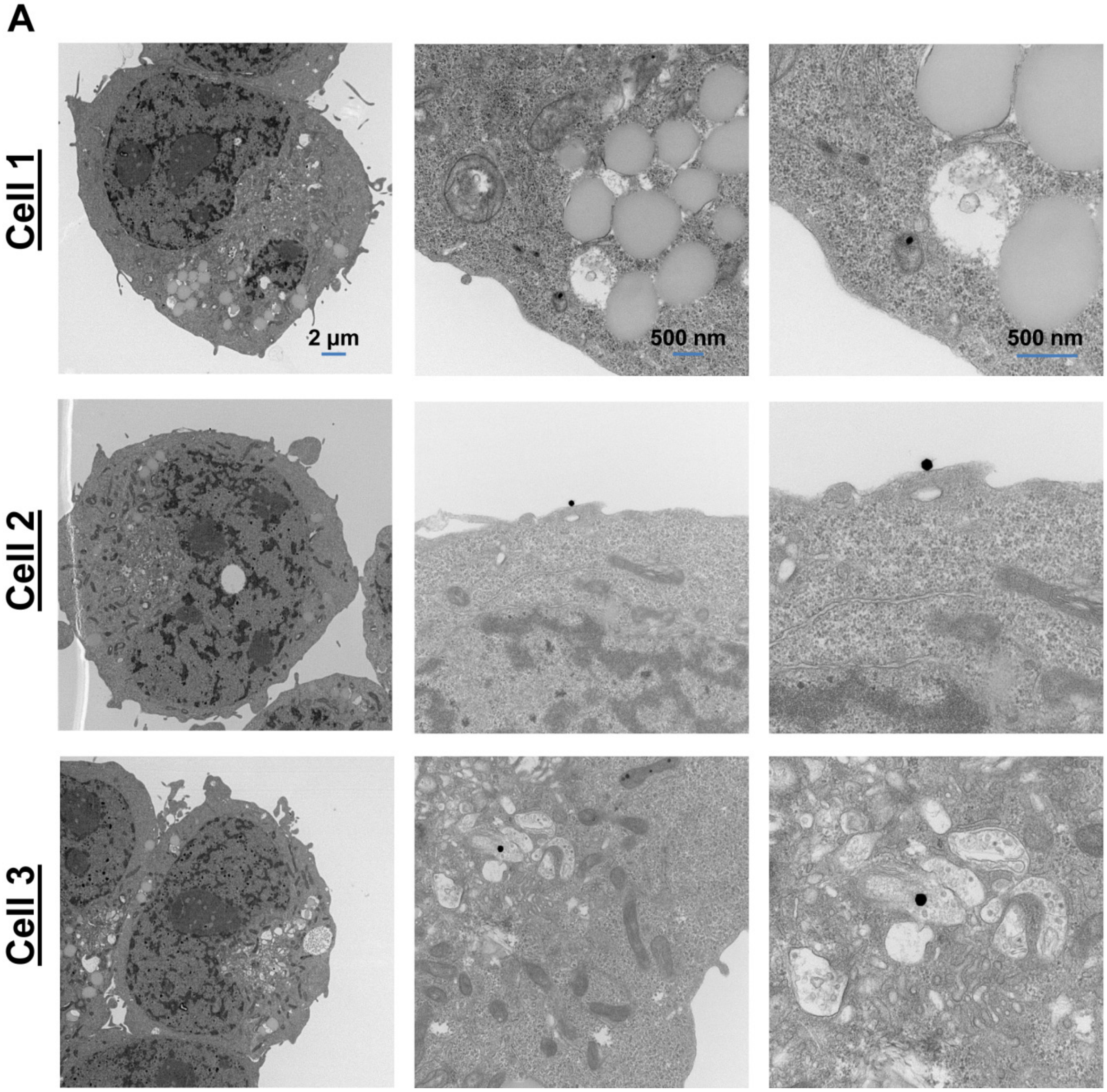

Figure S3 Additional TEM images of Sk-Br-3 cells following A) 2 h, B) 5 h, C) 10 h, or D) 24 h incubation with 24 µg/mL 100 nm AuNPs. TEM images of multiple cells are shown to highlight the diversity of NP distribution inside cells.

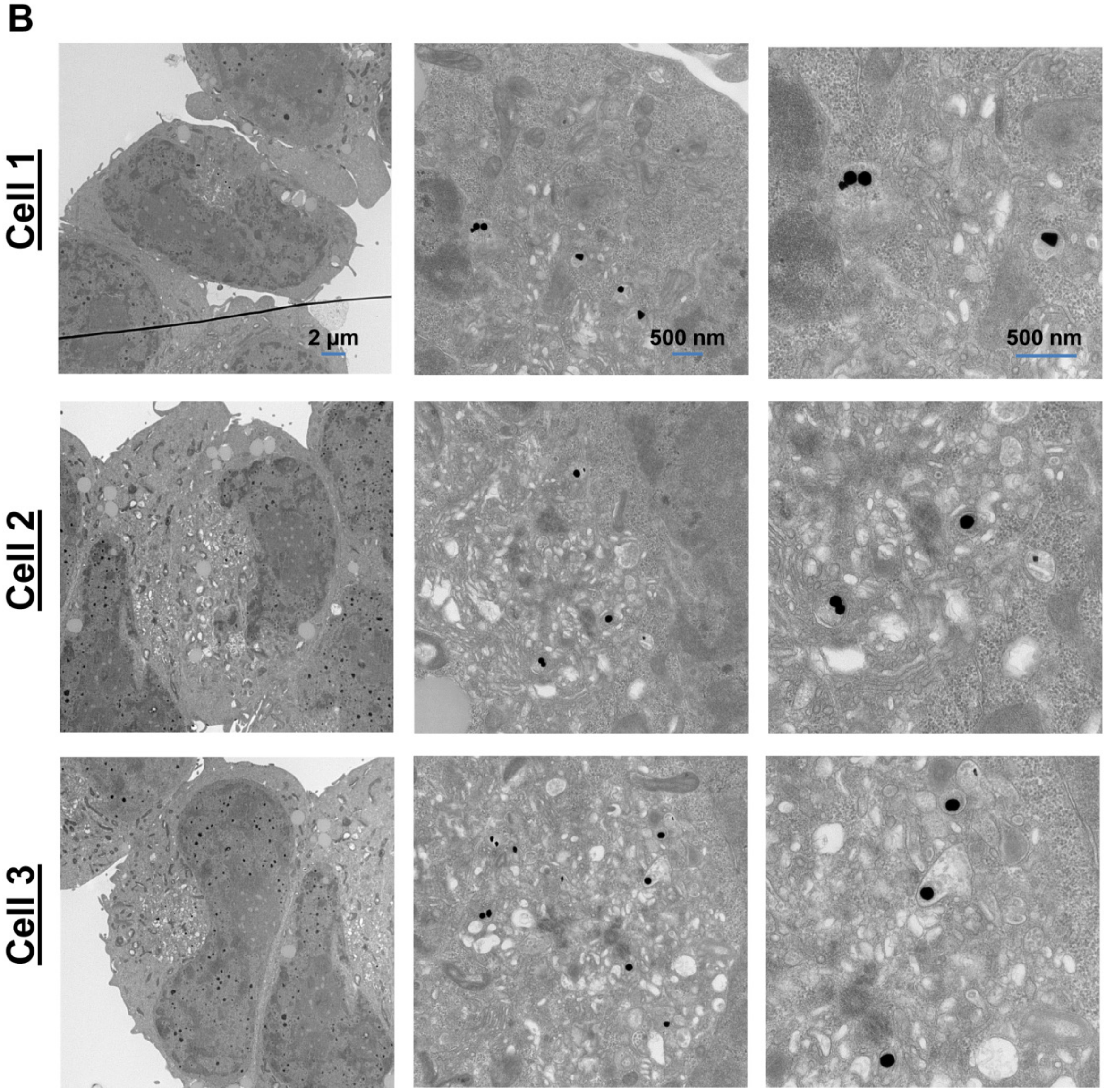

Figure S3 Additional TEM images of Sk-Br-3 cells following A) 2 h, B) 5 h, C) 10 h, or D) 24 h incubation with 24 µg/mL 100 nm AuNPs. TEM images of multiple cells are shown to highlight the diversity of NP distribution inside cells.

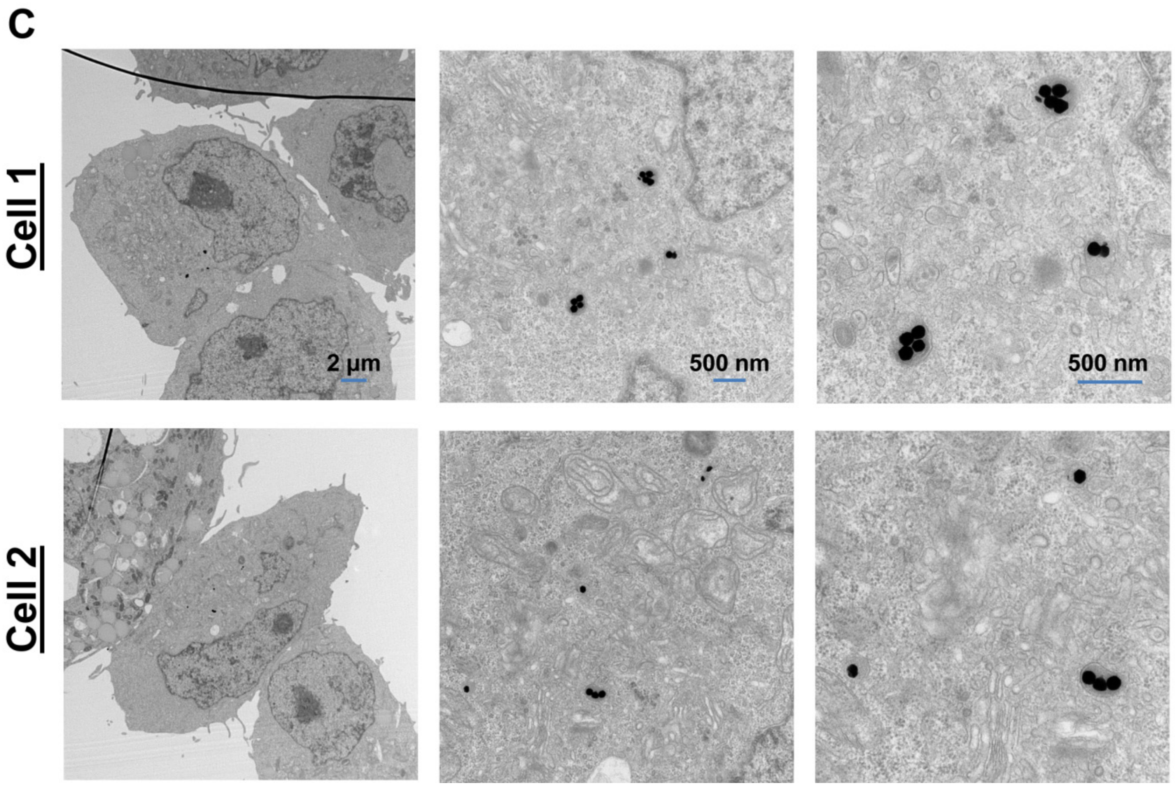

Figure S3 Additional TEM images of Sk-Br-3 cells following A) 2 h, B) 5 h, C) 10 h, or D) 24 h incubation with 24 µg/mL 100 nm AuNPs. TEM images of multiple cells are shown to highlight the diversity of NP distribution inside cells.

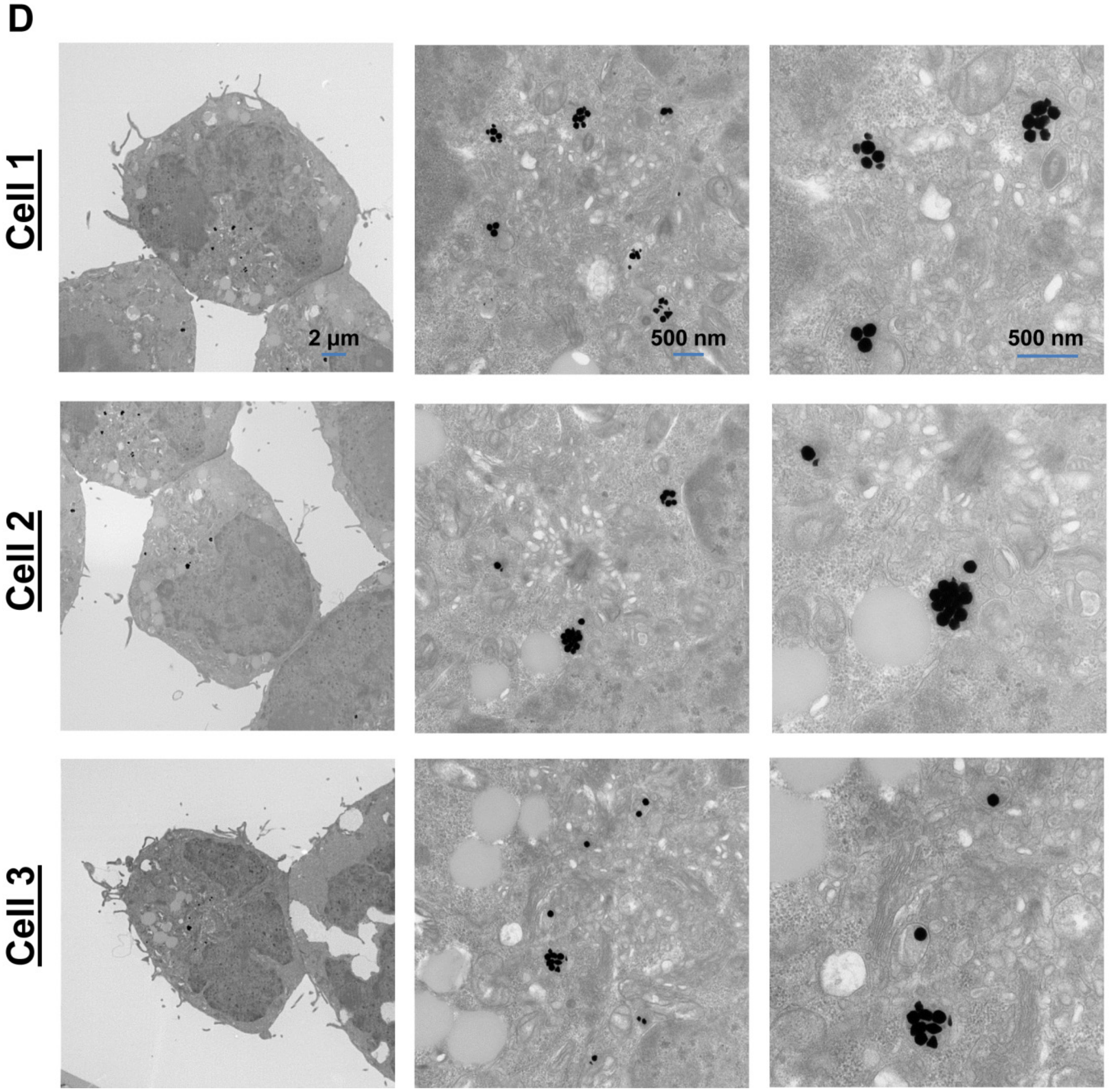

Figure S3 Additional TEM images of Sk-Br-3 cells following A) 2 h, B) 5 h, C) 10 h, or D) 24 h incubation with 24 µg/mL 100 nm AuNPs. TEM images of multiple cells are shown to highlight the diversity of NP distribution inside cells.

4. Cell viability results

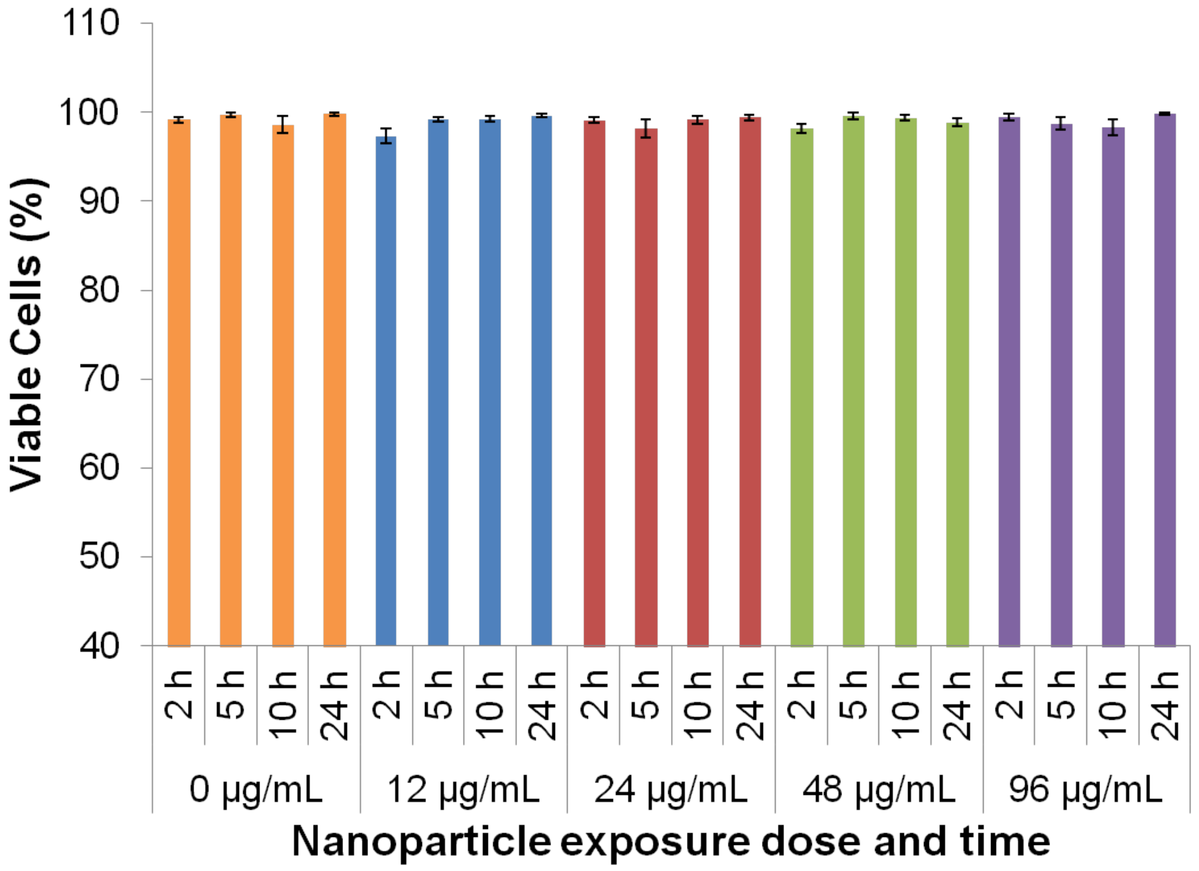

Figure S4 Cell viability determined by Live/Dead assay for cells exposed to 0, 12, 24, 48, or 96 µg/mL NPs for 2, 5, 10, or 24 h. Cells maintain viability following NP incubation at the NP exposure doses utilized, suggesting the plasma membrane is intact and spectral changes are not due to loss in membrane integrity. Error bars represent standard error of the mean for n=3.

5. Additional cellular TEM images: *varying exposure dose*

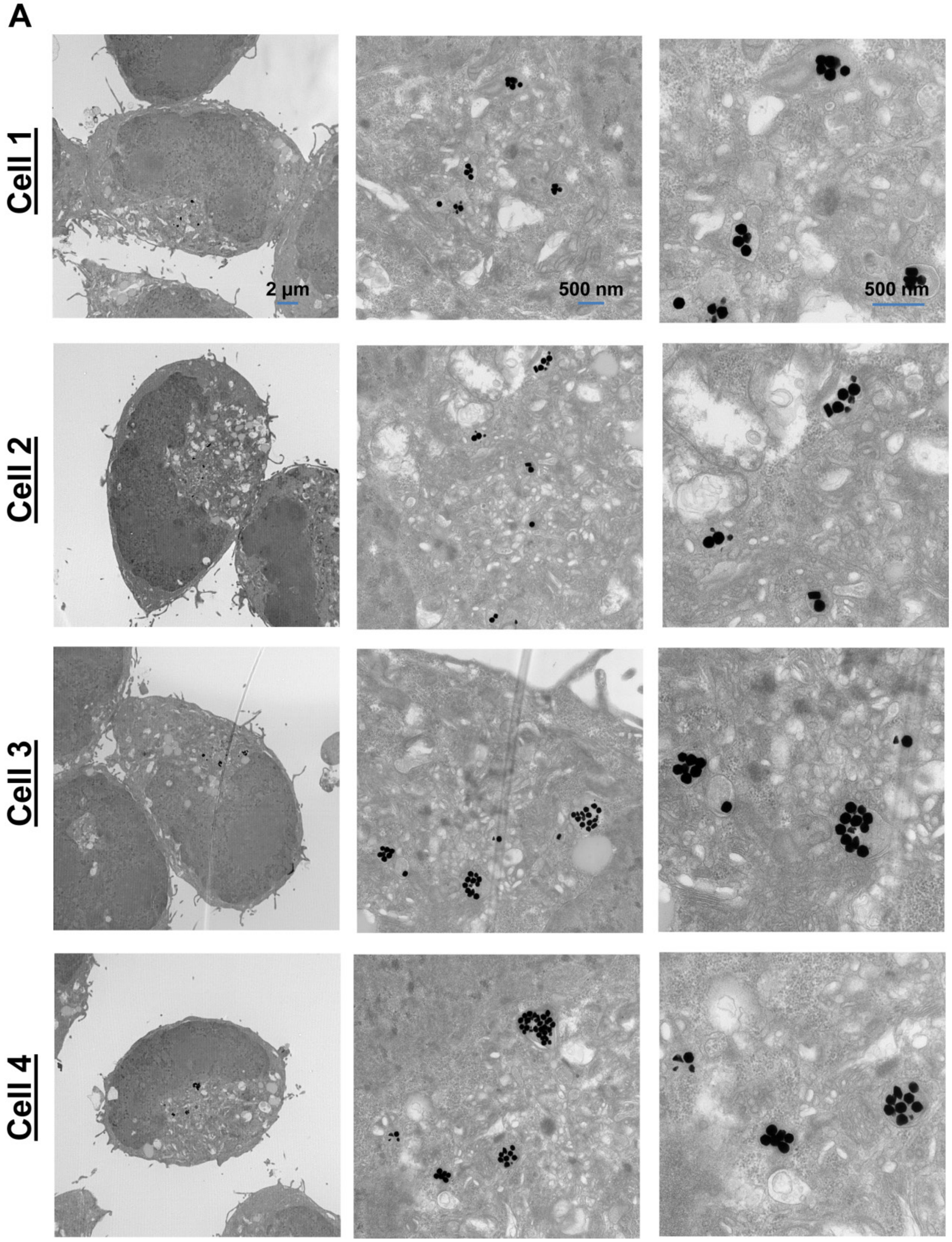

Figure S5 Additional TEM images of Sk-Br-3 cells following 24 h incubation with A) 12, B) 48, or C) 96 μg/mL 100 nm AuNPs. TEM images of multiple cells are shown to highlight the diversity of NP distribution inside cells.

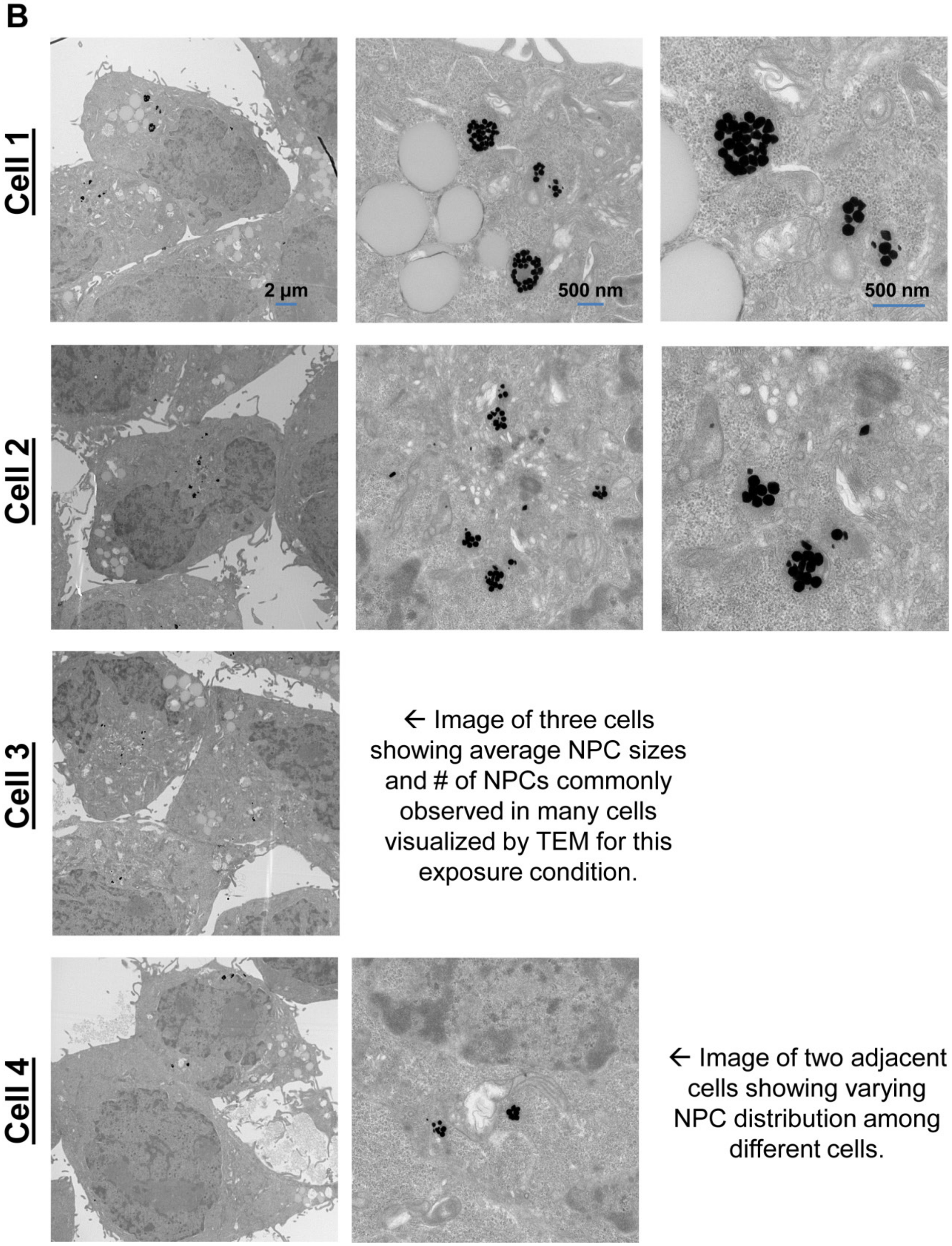

Figure S5 Additional TEM images of Sk-Br-3 cells following <u>24 h</u> incubation with A) 12, B) <u>48</u>, or C) 96 <u>µg/mL</u> 100 nm AuNPs. TEM images of multiple cells are shown to highlight the diversity of NP distribution inside cells.

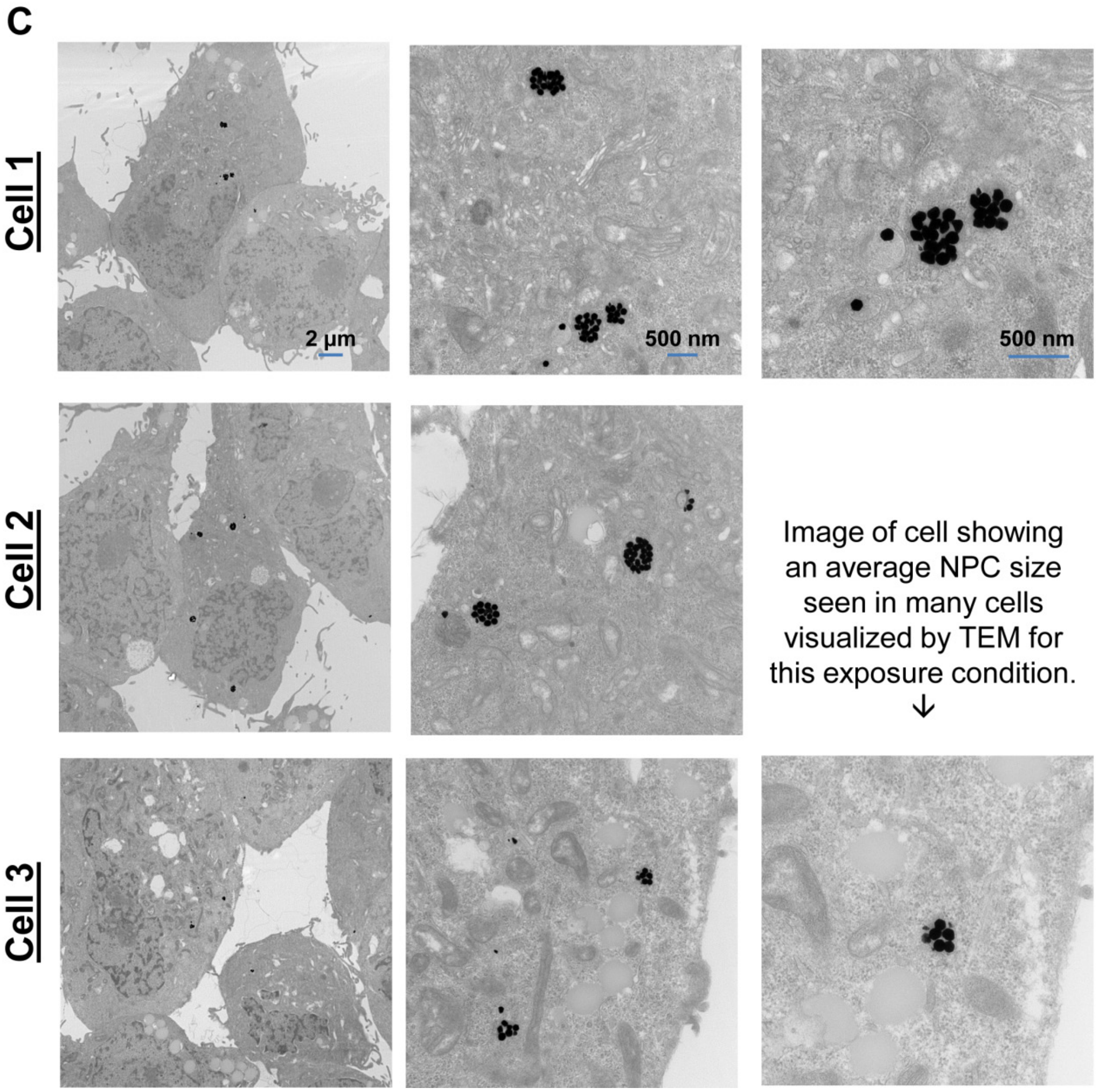

Figure S5 Additional TEM images of Sk-Br-3 cells following 24 h incubation with A) 12, B) 48, or C) 96 µg/mL 100 nm AuNPs. TEM images of multiple cells are shown to highlight the diversity of NP distribution inside cells.

6. Nanoparticle cluster ROI spectral peak wavelength and broadness measurements

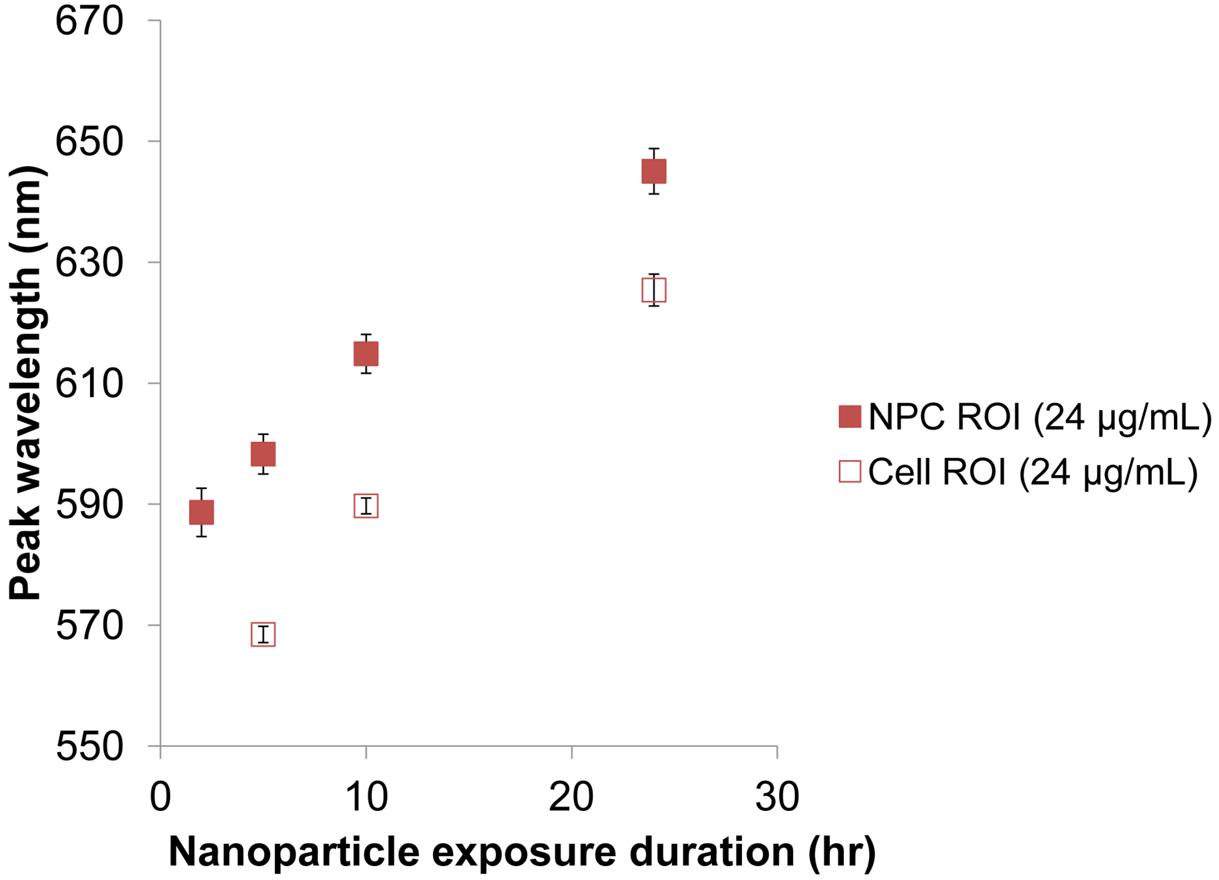

Figure S6 NPC ROI-derived spectral peak wavelengths for cells exposed to 24 µg/mL NPs for 2, 5, 10, or 24 h. For comparison, peak wavelengths of spectra extracted from cell ROIs of the same samples are plotted as open squares. Error bars denote the standard error of the mean for 56 to 180 NPC ROIs and 32 to 107 cell ROIs (representing the subset of cells containing sufficient signal for analysis out of the 64 to 111 cell ROIs that were defined; see pg. 17 of Additional File 1 for details).

While cell ROI spectra showed a symmetric distribution of peak wavelengths across the cell population (Figure 6 in main text), NPC ROI spectra had an asymmetric distribution of peak wavelengths that was positively skewed with a large portion of NPCs exhibiting spectral peaks at shorter wavelengths (580-600 nm) after 5-10 h NP exposure (Figure S7A in Additional File 1). This may be because at these early time points, a large proportion of the NPCs within cells contain only few NPs (Figure S3 in Additional File 1), and the small number of larger NPCs is responsible for the low frequency of NPC ROI spectra with longer peak wavelengths. By 24 h, the peak wavelengths of NPC ROIs were more equally distributed; indeed, cells in TEM images show a diversity ranging from 1-20 NPs per NPC and larger clusters are common (Figure S3 in Additional File 1).

As expected since NPC ROI spectra do not contain a large cell scattering component and are not integrated over multiple NPC ROIs exhibiting a variety of spectra, NPC ROI spectra were narrower than those of cell ROI spectra. Spectral width ranged from 48.7 ± 14.9 nm after 2 h NP exposure to 64.9 ± 24.5 nm after 24 h exposure (Figure S7B in Additional File 1).

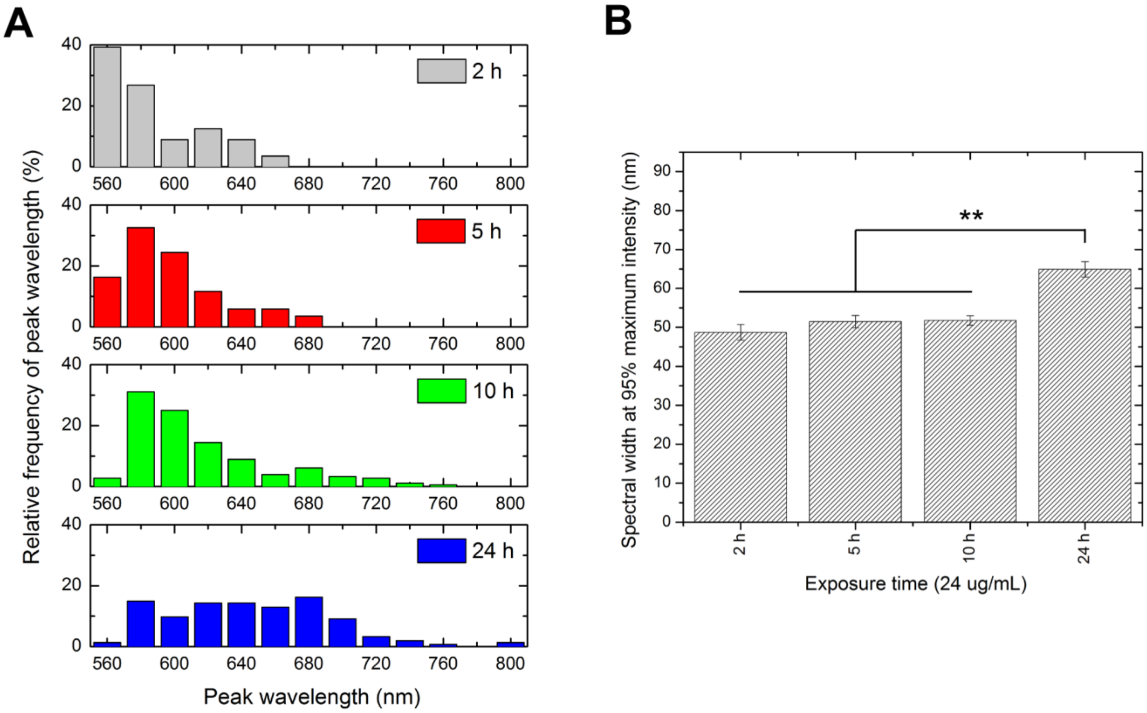

Figure S7 A) Distribution of peak wavelengths of NPC ROI spectra following cellular exposure to 24 μg/mL AuNPs for 2, 5, 10, or 24 h. B) Mean spectral width at 95% of maximum intensity with increasing exposure duration. Error bars denote standard error of the mean for 56 to 180 NPC ROIs. ** denotes $p<0.01$.

7. Additional darkfield HS imaging methods and supporting rationale

Hyperspectral images were taken at both the slide plane, which coincides with the cell's basal plane of attachment to the slide, as well as 5 μm above this plane. As seen in the slide plane images (left column), NPs are visible in areas on the slide where no cells are present. NPs from the NP suspension in culture medium deposit onto the slide surface, similar to what has been observed by other studies [1-3]. Spectra for analyses were extracted from the hyperspectral images taken at 5 μm above the slide plane in order to obtain spectra primarily arising from intracellular NPs. While light scattering interactions with other planes can still contribute to the spectra, analyzing spectra from this consistent focal plane position within cells allows us to capture spectra dominated by contributions arising from NP clusters within the cell.

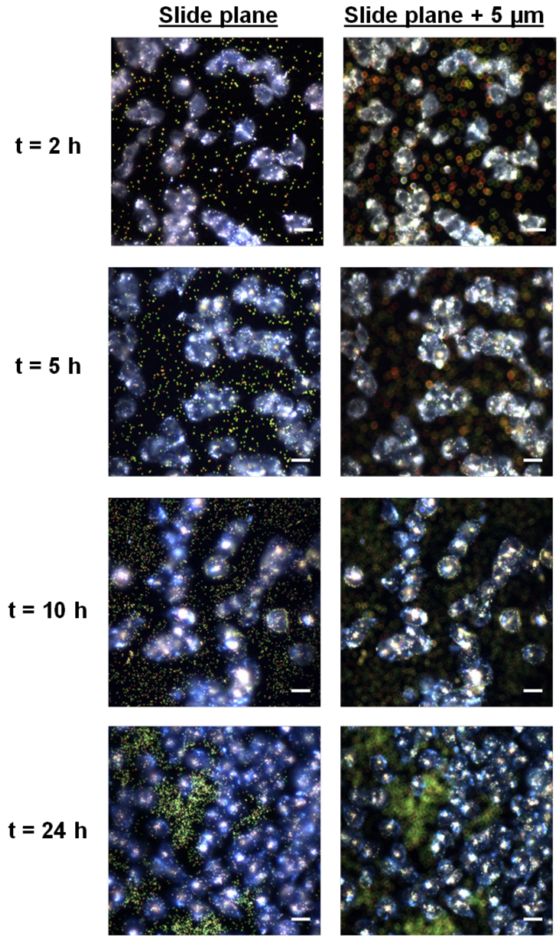

Figure S8 Representative HS images taken at the slide plane (left column) or 5 μm above the slide plane (right column images). Spectra were extracted from HS images taken 5 μm above the slide plane to focus analysis on the optical response from intracellular NPs. Scale bar denotes 20 μm.

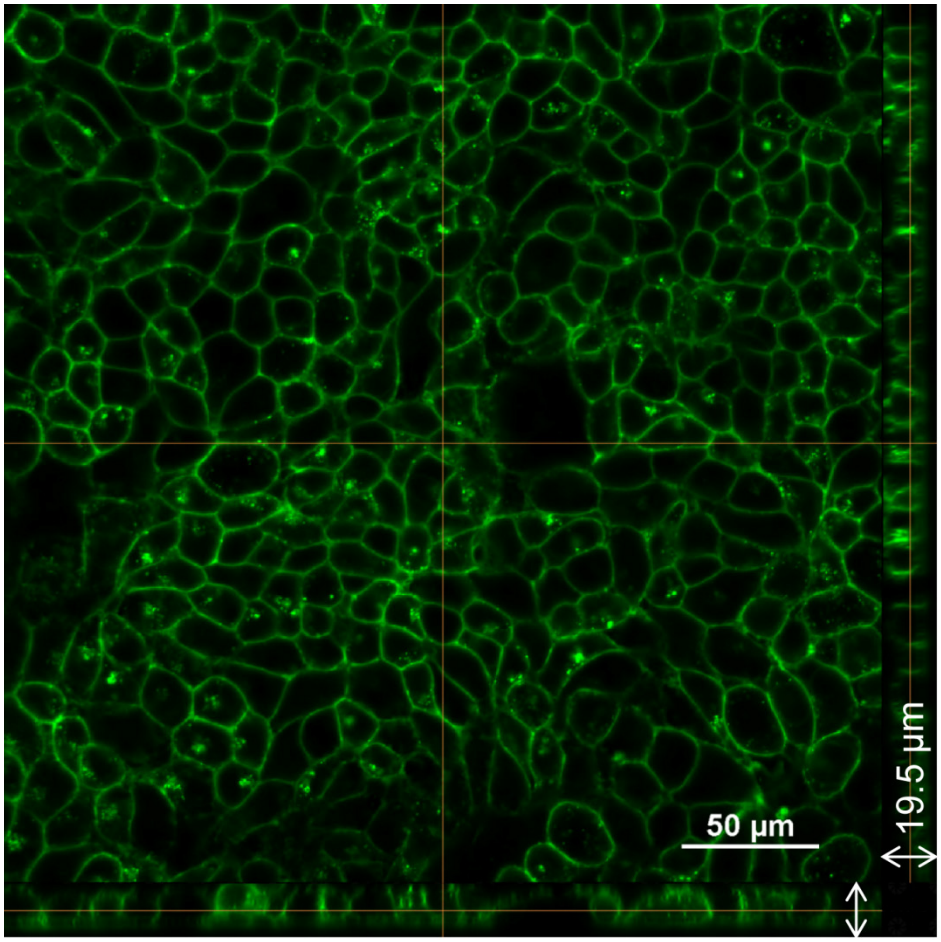

Figure S9 Confocal z-stack image of Sk-Br-3 cells on chamber slide to confirm cell height. The plasma membrane was labeled with wheat germ agglutinin (WGA)-Oregon Green conjugate. The X-Z and Y-Z slice views depict cross sections of the cell, showing that cell height ranges from 10~20 µm. When we moved across cell focal planes using a z focus drive in the CytoViva HS Imaging System setup at 40x magnification (~<1 µm depth of field), we also found that cell height ranged from 10~20 µm.

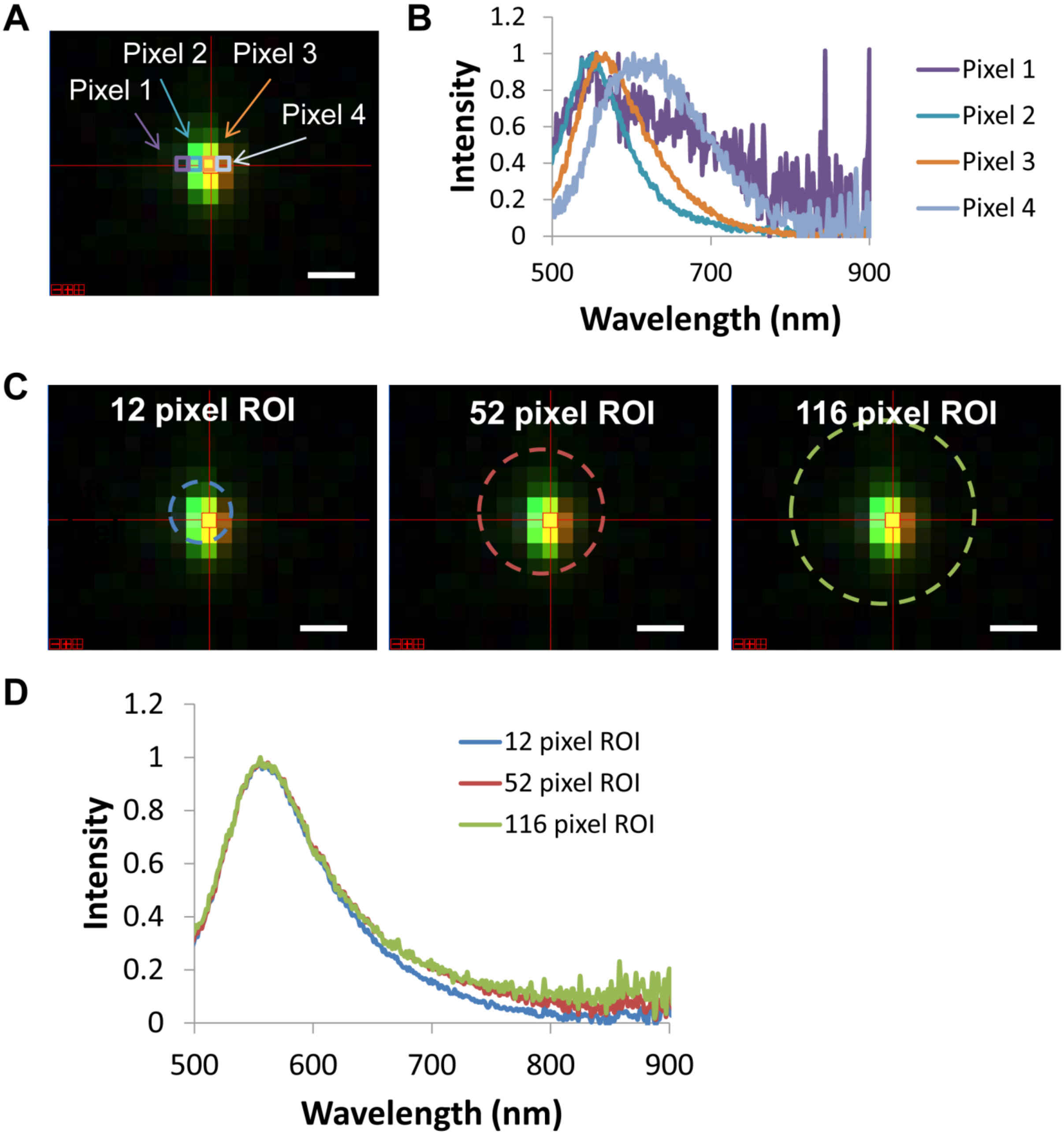

Figure S10 Impact of how region of interest (ROI) is defined on the scattering spectra for 100 nm AuNP. A) HS image of AuNP with ROI defined as single pixels. B) Corresponding spectra extracted from the single pixels defined in A. Spectra vary when extracted from a single pixel of the NPC, depending on the pixel selected. C) HS image of 100 nm AuNP with ROI defined as a 12 pixel, 52 pixel, or 116 pixel ellipse ROI around the NPC. D) Corresponding spectra extracted from multi-pixel ellipse ROIs shown in C. By defining a multi-pixel ellipse ROI around the NPC, variations are averaged out and ROIs can be defined consistently. Scale bar represents approximately 1 µm.

8. HS image analysis and sample sizes

In order to measure HS image from cells without bias toward cells with greater NP uptake, cell ROIs were defined around all cells in each HS image (except when the boundaries of the cell could not be unambiguously defined). Because of this, the sample size n varied. For Figure 4 in the main text, 63 to 125 cell ROIs were defined per condition. However, because some cell ROI spectra were dominated by cell scattering and a peak wavelength could not be calculated, the resulting sample size actually ranged from n=12 to 113 cell ROI spectra. All $n \geq 63$ except for the 96 µg/mL at 2 h, 12 µg/mL at 5 h, and 24 µg/mL at 5 h exposure dose/time conditions. For these conditions, although 74, 125, and 111 cell ROI spectra were measured, n = 35, 12, and 32, respectively because only this subset of spectra had sufficient NP signal for peak wavelength analysis (the cell scattering spectra dominated over the minimal NP scattering at these early time points).

Below is a table that summarizes the # of cell ROI spectra measured for each condition ("# ROIs") and the # of spectra with sufficient signal for calculation of peak wavelength ("n"). The % of cells with sufficient NP signal is plotted in Figure 7 of the main text. Criteria for determining if cell ROI spectra had sufficient NP signal for analysis is described in Methods.

Table S1. Summary of the # cell ROI spectra that were defined for each condition and n cell ROI spectra that were included in peak wavelength analyses. Peak wavelengths could only be calculated from cell ROI spectra with sufficient NP signal. Thus, $n \leq$ # ROIs, especially at early time points where cell ROI spectra of a majority of cells were dominated by cell scattering.

	**12 µg/mL**		**24 µg/mL**		**48 µg/mL**		**96 µg/mL**	
**Time**	**n**	**# ROIs**	**n**	**# ROIs**	**n**	**# ROIs**	**n**	**# ROIs**
**2 h**	0	63	0	65	0	64	35	74
**5 h**	12	125	32	111	69	98	90	91
**10 h**	83	93	63	64	90	90	77	77
**24 h**	113	113	107	109	96	96	91	91

9. Electric field calculation setup

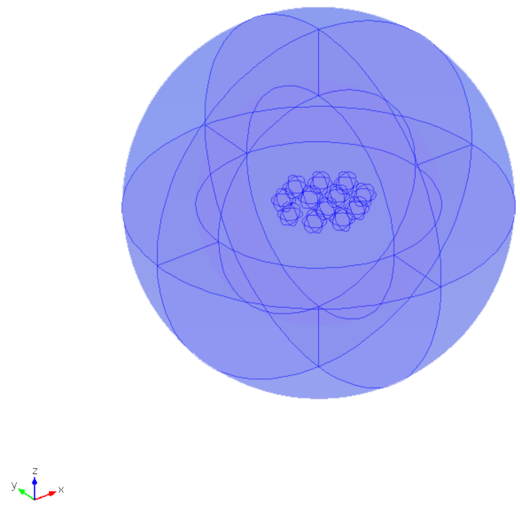

Figure S11 A typical simulation geometry with the E field polarized in *x* in plane with the NPC and propagating through the NPC in *z*.

Table S2. Coordinates of the center positions of AuNPs in each simulation. Diameter d = 100 nm, g = 5 nm.

	AuNP 1	AuNP 2	AuNP 3	AuNP 4	AuNP 5	AuNP 6	AuNP 7	AuNP 8	AuNP 9	AuNP 10	AuNP 11	AuNP 12
monomer	(0,0,0)											
dimer	(-d/2-2g, 0,0)	(d/2+2g,0,0)										
trimer	(0,d/2+g,0)	(0,-d/2-g,0)	(d-g,0,0)									
heptamer	(0,0,0)	(-d/2-g/2,d-2g,0)	(d/2+g/2,d-2g,0)	(-d-g,0,0)	(d+g,0,0)	(d/2+g/2,-d+2g, 0)	(-d/2-g/2,-d+2g, 0)					
dodecamer	(0,d/2+g,0)	(0,-d/2-g,0)	(d-g,0,0)	(0,3d/2+2g,0)	(d,d+5g,0)	(2d-3g,d/2+2g,0)	(2d-3g,-d/2-2g, 0)	(d,-d-5g,0)	(0,-3d/2-2g,0)	(-d,-d-g,0)	(-d-9g,0,0)	(-d,d+g,0)

d = 100 nm
g = 5 nm

10. References for Additional File 1

1. Lesniak A, Salvati A, Santos-Martinez MJ, Radomski MW, Dawson KA, Aberg C: **Nanoparticle adhesion to the cell membrane and its effect on nanoparticle uptake efficiency.** *Journal of the American Chemical Society* 2013, **135:**1438-1444.

2. Lesniak A, Fenaroli F, Monopoli MR, Aberg C, Dawson KA, Salvati A: **Effects of the presence or absence of a protein corona on silica nanoparticle uptake and impact on cells.** *ACS Nano* 2012, **6**.

3. dos Santos T, Varela J, Lynch I, Salvati A, Dawson KA: **Quantitative assessment of the comparative nanoparticle-uptake efficiency of a range of cell lines.** *Small* 2011, **7**.
